# Resveratrol Sensitizes Colorectal Cancer Cells to Cetuximab by Connexin 43 Upregulation-Induced Akt Inhibition

**DOI:** 10.3389/fonc.2020.00383

**Published:** 2020-04-07

**Authors:** Yijia Wang, Wenhong Wang, Xiaojing Wu, Chunjun Li, Yaping Huang, Huiyan Zhou, Yu Cui

**Affiliations:** ^1^Laboratory of Oncologic Molecular Medicine, Tianjin Union Medical Center, Tianjin, China; ^2^State Key Laboratory of Medicinal Chemical Biology, NanKai University, Tianjin, China; ^3^Beijing Enmin Technology Co. Ltd, Beijing, China

**Keywords:** Akt, cetuximab, colorectal cancer, connexin 43, resveratrol

## Abstract

Cetuximab is a monoclonal antibody that acts as an anti-epidermal growth factor receptor (EGFR) agent. Cetuximab inhibits the phosphorylation and activation of EGFR and blocks downstream signal pathways of EGF/EGFR, including Ras-Raf-MAPK and PI3K-Akt pathways. Akt activation is an important factor in cetuximab resistance. It has been reported that resveratrol and connexin 43 regulate Akt in different ways based on tissue type. Since connexin 43 interacts with Akt, and resveratrol is known to upregulate connexin 43, we investigated whether resveratrol can sensitize colorectal cancer cells to cetuximab via connexin 43 upregulation. Our work confirmed that resveratrol increases the inhibition of growth by cetuximab *in vitro* and *in vivo*, upregulates connexin 43 expression and phosphorylation, increases gap junction function, and inhibits the activation of Akt and NFκB in parental or cetuximab-treated parental HCT116 and CT26 cells. Resveratrol did not exhibit these effects on connexin 43-shRNA transfected cells, so connexin 43 upregulation may contribute to Akt inhibition in these cells. Given these data, resveratrol may sensitize colorectal cancer cells to cetuximab via upregulating connexin 43 to inhibit the Akt pathway.

## Introduction

Epidermal growth factor receptor (EGFR) is widely expressed by both normal and malignant epithelial cells. EGFR contributes to metastasis, angiogenesis, proliferation, and inhibition of apoptosis in tumor biology. EGFR is frequently overexpressed in various cancers and has emerged as an attractive target for cancer chemotherapy (1). Cetuximab is a recombinant chimeric human–mouse monoclonal antibody that binds to the extracellular ligand-binding domain of EGFR with high affinity. Cetuximab is an EGFR inhibitor and disturbs other endogenous ligands binding to EGFR to inhibit the phosphorylation and activation of EGFR (1). Cetuximab blocks the downstream signaling pathways of receptor tyrosine kinase, which include Ras-Raf-MAPK and PI3K-Akt. Disruptions in these two pathways are a major source of cetuximab resistance, such as those caused by mutations in NRAS, KRAS, BRAF, and PI3KCA. Furthermore, Akt activation is also an important factor in cetuximab resistance. The PI3K-Akt pathway can be activated by phosphorylation of EGFR, so it is inhibited by cetuximab in most cases. However, there are other mechanisms to activate Akt besides the phosphorylation of EGFR, and resistance emerges if Akt maintains activation during cetuximab therapy. Some research has found that cetuximab resistance relates to Akt activation in many cell lines, phosphorylated Akt (p-Akt) still occurs at a certain level after cetuximab treatment in these cells, and inhibiting the Akt pathway with other reagents is an effective strategy to sensitize these cell lines to cetuximab (2).

Resveratrol (3,5,4′-trihydroxy-trans-stilbene) is a polyphenolic compound that has been shown to have various anticancer, anti-inflammatory, and antioxidant activities (3, 4). Several studies have shown that resveratrol downregulates p-Akt in lung (5) or breast cancer cell lines (6), but resveratrol also increases p-Akt to prevent paclitaxel-induced neuropathic pain (7) or prevent ischemia-reperfusion injury (8). It is reported that combined treatment with resveratrol and 5-FU reduced p-Akt in colorectal cancer (9). Combined treatment with resveratrol sensitized colorectal cancer cells to 5-FU, inducing a further increase in oxidative stress, which was linked to the inhibition of AKT and STAT3 proteins, which are known to have oncogenic potential in colorectal carcinomas (10). Resveratrol in combination with forskolin has phosphodiesterase 4D inhibitory effects to inhibit Akt/mTOR signaling in colorectal cancer cells (11). But there is still a lack of research on the effect and mechanism of resveratrol used as a single agent to regulate p-Akt in colorectal cancer. Resveratrol exhibits antitumor activity when used alone (12) or in combination with other drugs (13), but whether resveratrol can enhance cetuximab cytotoxicity by inhibiting p-Akt in cancer cells remains unknown.

In addition, resveratrol upregulates connexin 43 (Cx43), a crucial component of gap junctions in human cells (14). It is reported that Cx43 up- or downregulates p-Akt in different cells. For example, Cx43 inhibits p-Akt through protein–protein interactions in glioblastoma (15) but contributes to p-Akt in cardiomyocytes (16). p-Akt also upregulates (17) or downregulates (18) the phosphorylation of Cx43 (p-Cx43), depending on the cell type. Phosphorylation is a necessary step for Cx43 to form gap junctions (19), which sensitizes cancer cells to many antitumor drugs (20). However, the nature of the interaction between p-Cx43 and p-Akt in colorectal cancer cells, and whether this interaction affects the cytotoxicity of cetuximab, are still unknown.

In this study, two colorectal cancer cell lines, HCT116 and CT26, were used to investigate the effect of resveratrol treatment on the cytotoxicity of cetuximab *in vitro* and *in vivo*. The influence of resveratrol on p-Akt in colorectal cancer cells, whether this influence affects cetuximab resistance, and how resveratrol-induced Cx43 variation contributes to p-Akt, are all examined in this work.

## Materials and Methods

### Reagents and Antibodies

All cell culture media, trypsin, and antibiotics were purchased from Gibco (Grand Island, NY, USA), and fetal bovine serum (FBS) was purchased from HyClone (Logan, UT, USA). Rabbit anti-Cx43 antibody, rabbit anti-phospho-Cx43 (pSer^368^) antibody, rabbit anti-EGFR antibody, rabbit anti-phospho-EGFR (pTyr^1092^) antibody, rabbit anti-PI3K p85-α antibody, rabbit anti-phospho-PI3K p85-α (pTyr^607^) antibody, rabbit anti-Akt antibody, rabbit anti-phospho-Akt (pSer^473^) antibody, rabbit anti-mTOR antibody, rabbit anti-phospho-mTOR (pSer^2448^) antibody, rabbit anti-IKKα antibody, rabbit anti-IκBα antibody, mouse anti-nuclear factor kappa B (anti-NFκB) p65 antibody, rabbit anti-p38 MAPK antibody, rabbit anti-phospho-p38 MAPK (pThr^179^ + Tyr^181^) antibody, mouse anti-β-actin antibody, rabbit anti-Histone H3 antibody, goat anti-rabbit IgG-peroxidase, goat anti-mouse IgG-peroxidase, goat anti-mouse IgG FITC, goat anti-rabbit IgG TRITC, DAPI, Calcein-AM, CM-Dil, resveratrol, carbenoxolone disodium, and methyl thiazolyl tetrazolium (MTT) were purchased from Sigma-Aldrich (St. Louis, MO, USA). Cetuximab was purchased from Merck KgaA (Darmstadt, Germany). Immobilon membranes were purchased from Merck Millipore (Bedford, MA, USA). RIPA lysis buffer, bicinchoninic acid reagents, and ECL Plus substrate were purchased from CWBio (Beijing, China). Rabbit IgG, nuclear and cytoplasmic protein extraction kit, and crystal violet were purchased from Beyotime (Shanghai, China). Cx43 shRNA plasmid (sc-29276-SH and sc-35091-SH), control shRNA plasmid, Plasmid Transfection Reagent, Plasmid Transfection Medium, mouse anti-Cx43 antibody, and protein A/G PLUS-Agarose were purchased from Santa Cruz Biotechnology (Dallas, TX, USA). pTARGET Mammalian Expression Vector system was purchased from Promega Corporation (Madison, WI, USA).

### Cell Lines

HCT116 and CT26 cell lines were purchased from the Shanghai Institutes for Biological Sciences, Chinese Academy of Sciences (Shanghai, China). HCT116 is a human colorectal cancer cell line. CT26 is a murine colon adenocarcinoma cell line derived from N-nitroso-N-methylurethane-treated Balb/c mice. All cells were cultured in an RPMI 1640 medium supplemented with 10% FBS, 100 μg/ml streptomycin, and 100 U/ml penicillin.

### Knockdown and Transfection of Cx43

For knockdown of Cx43, HCT116 and CT26 cells were transfected with Cx43 shRNA (sc-29276-SH for HCT116 and sc-35091-SH for CT26) or control shRNA plasmids using the shRNA transfection reagent according to the manufacturer's instructions. Briefly, for each transfection, we diluted 10 μl of resuspended plasmid DNA (1 μg plasmid DNA) into a 90-μl plasmid transfection medium (sc-108062) to form solution A. We then diluted 4 μl of a plasmid transfection reagent (sc-108061) with a plasmid transfection medium to bring the final volume to 100 μl to form solution B. We added solution A directly to solution B and incubated the mixture for 20 min at room temperature. We then gently washed the cells twice with 2 ml of the transfection medium, and a 0.8-ml plasmid transfection medium was added to each well for transfection; 200 μl of the complex (solution A + solution B) was added dropwise to each well. Cells were incubated for 7 h at 37°C in a 5% CO_2_ incubator. After incubation, we added 1 ml of a normal growth medium containing 20% FBS and 2 μg/ml puromycin to the cells, and cells were incubated for an additional 24 h at 37°C in a 5% CO_2_ incubator. We aspirated the medium and replaced it with a normal growth medium. The cells were used for assays after 72-h incubation.

The method of Cx43 transfection was carried out as previously described. Briefly, cDNA of the human Cx43 coding region was inserted into the pTARGET vector. The constructed expression vector was transfected to cells by lipofection. Cx43 expression in transfected cells is identified by western blotting.

### Resveratrol and Cetuximab Treatment

Cells were treated with various concentrations of resveratrol and cetuximab, in four different groups: (1) cells without drug treatment were the control group; (2) cells treated with resveratrol alone for 24 h; (3) cells treated with cetuximab alone for 24 h; and (4) cells treated with a mixture of resveratrol and cetuximab for 24 h. Furthermore, cells pretreated with 50 μM carbenoxolone for 12 h were also used for the assay. Cell viability was measured by MTT assay after treatment. The rate of cell growth inhibition in each well was calculated by defining the absorption of non-treated cells (control) as 100%. Measurements were performed in triplicate.

### Western Blotting and Immunoprecipitation

According to the results of the section Resveratrol and Cetuximab Treatment, cells treated with 10 μg/ml cetuximab and/or 5 μg/ml resveratrol were used for experiments; 10^6^ cells were lysed in a 500-ml RIPA lysis buffer supplemented with protease inhibitors for 20 min at 4°C. The lysates were centrifuged, and bicinchoninic acid reagents were used to quantitate the protein content.

Western blotting was carried out as previously described (21). Briefly, protein samples were suspended in an SDS loading buffer. After boiling, 10 μg protein was separated by SDS-PAGE and then transferred to Immobilon PVDF membranes, using the semi-dry blotting method. The membranes were probed with antibodies using standard techniques. Finally, the protein bands were visualized using ECL Plus and exposed film. Each assay was carried out in triplicate.

To detect NF-κB expression in nuclei, nuclear proteins were extracted from cells using a nuclear and cytoplasmic protein extraction kit according to the manufacturer's instructions. In brief, 200 μl cytosolic protein extraction agent A with 1 mM phenylmethyl sulfonylfluoride (PMSF) was added to 20 μl harvested cells at 4°C. After incubating on ice for 15 min, 10 μl cytosolic protein extraction agent B was added. Following vigorous vortexing for 5 s, the tubes were incubated on ice for 1 min. Then the tubes were vortexed vigorously for 5 s once more and centrifuged at 15,000 × g for 5 min at 4°C. Nuclear proteins were contained in the insoluble pellet, which was resuspended in a 50-μl nuclear protein extraction agent with 1 mM PMSF. Then the tubes were vortexed vigorously for 20 s and incubated on ice for 30 min. During this incubation, the tubes were vortexed six times every 5 min, each time for 15 s. Tubes were then centrifuged at 15,000 × g for 10 min at 4°C. Nuclear proteins were in the supernatant and were transferred to a fresh tube for measurement.

The interaction between Cx43 and Akt was analyzed by immunoprecipitation (IP) as previously described (22). Briefly, 4 ml cell lysate was incubated with 1 μg rabbit IgG and 20 μl protein A/G PLUS-Agarose at 4°C for 30 min. The mixture was centrifuged at 1,000 × g for 5 min at 4°C to collect the supernatant. The supernatant was collected and the protein amounts were standardized; 300 μg total cellular protein was incubated with 1 μg Akt or Cx43 antibody (or 1 μg rabbit IgG as negative control) for 1 h at 4°C, and then 20 μl protein A/G PLUS-Agarose was added to the mixture and incubated overnight at 4°C. The immunoprecipitates were collected by centrifugation at 1,000 × g for 5 min at 4°C and washed with PBS. Then the precipitates were subjected to western blot analysis with primary antibody against Cx43 or Akt, respectively.

### “Parachute” Dye-Coupling Assay

Gap junction (GJ) function was examined using the “Parachute” dye-coupling assay as described by Wang et al. (23). Briefly, cells were seeded in six-well plates and grown to 80–100% confluency. CM-Dil and Calcein-AM were used to determine the GJ function. CM-Dil is a red fluorescent dye that cannot spread to coupled cells. Calcein-AM is a green fluorescent dye that can spread to adjacent cells through GJ. Calcein-AM (10 μg/ml) and CM-Dil (5 μg/ml) were used to stain each well of donor cells for 30 min. Donor cells were then trypsinized and seeded onto a monolayer of receiver cells grown in another well. The ratio of donor to receiver cells was 1:150. Cells were incubated at 37°C for 4 h to form the GJ between donor and receiver cells. Fluorescence was detected using a fluorescence microscope. Red fluorescence of CM-Dil indicated the location of donor cells. The green fluorescence of Calcein-AM was used to calculate the number of illuminant receiver cells around each donor cell. This value was used to evaluate the GJ function. Each assay was carried out five times.

### Immunocytochemistry Analysis

Immunofluorescence staining was used to analyze Cx43 and Akt. The procedure was carried out as described previously (21). In brief, cells were washed twice with PBS, and fixed in 10% formalin at room temperature for 20 min, treated with 0.5% Triton X-100 for 5 min at 4°C followed by overnight blocking in 5% normal goat serum at 4°C. Following several PBS washes, slides were incubated with a mouse anti-Cx43 antibody or rabbit anti-Akt antibody for 1 h at 37°C. After washing with PBS, the slides were incubated with FITC-conjugated goat anti-mouse antibody and TRITC-conjugated goat anti-rabbit antibody and DAPI for 1 h at 37°C, followed by several washes in PBS. Finally, the slides were sealed then photographed immediately using a fluorescence microscope (Olympus, Tokyo, Japan).

### Subcutaneous Tumor Model

A subcutaneous tumor model was used to evaluate the inhibitory effect of resveratrol and cetuximab on tumors *in vivo*. Briefly, approximately 1 × 10^6^ parental or shRNA transfected CT26 or HCT116 cells were injected subcutaneously into the flank of 5 mice per group (6-week-old Balb/c females for CT26 and nu/nu nude mice for HCT116). An intraperitoneal injection of 2 mg/kg cetuximab and/or 1 mg/kg resveratrol was administered once a day for 14 days. Mice were sacrificed 15 days after cell implantation. Tumors were removed and weight was measured using electronic balance. All animal experiments complied with the National Institutes of Health guide for the care and use of laboratory animals (NIH Publications No. 8023, revised 1978).

### Statistical Analysis

All data represent mean ± SEM. One-way ANOVA was used to evaluate the significance of changes between different groups using SPSS software. *P* < 0.05 was considered statistically significant.

## Results

### Cx43 Expression Is Inhibited by Cx43-shRNA Transfection and Upregulated by Cx43-pTARGET Vector Transfection

**Figure 1:**
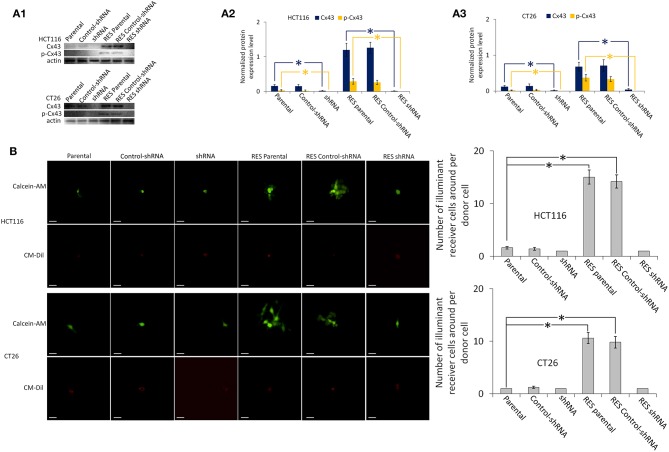
Cx43 expression and GJ function of HCT116 and CT26 cells, including parental, control-shRNA, and Cx43-shRNA transfected cells. RES, resveratrol-treated cells. All other groups were compared with the Parental group using one-way ANOVA. *P < 0.05 represents a significant difference from values in the Parental group. SEMs are shown as error bars. **(A1)** Western blot images of Cx43 and phospho-Cx43 (Ser-368). β-Actin is used as a loading control. The full-length blots are shown as Figure S7A. **(A2,A3)** Bar diagrams of densitometric analysis. Protein expression level is normalized by β-actin. **(B)** GJ function analysis by “Parachute” dye-coupling assay. Scale bars are 20 μm. Columns show the mean ± SEM. All images of “Parachute” dye-coupling assay are shown as Figure S1.

Colorectal cancer cells have a relatively low expression level of Cx43 compared with normal cells, and especially lack Cx43 expression on the membrane, so that the GJ function in these cancer cells is severely reduced (23). Our results (Figure 1 and Figure S1) showed that parental HCT116 and CT26 have a low expression level of Cx43, and that Cx43 expression is almost completely inhibited by Cx43-shRNA transfection. Neither parental nor transfected cells have the GJ function. Figure S2 showed that Cx43-pTARGET transfection increased Cx43 expression in CT26 and HCT116 cells. Full-length blots of Figure S2 are shown in Figure S7B.

### shRNA Transfection Decreases the Synergistic Effect of Combined Resveratrol and Cetuximab

**Figure 2:**
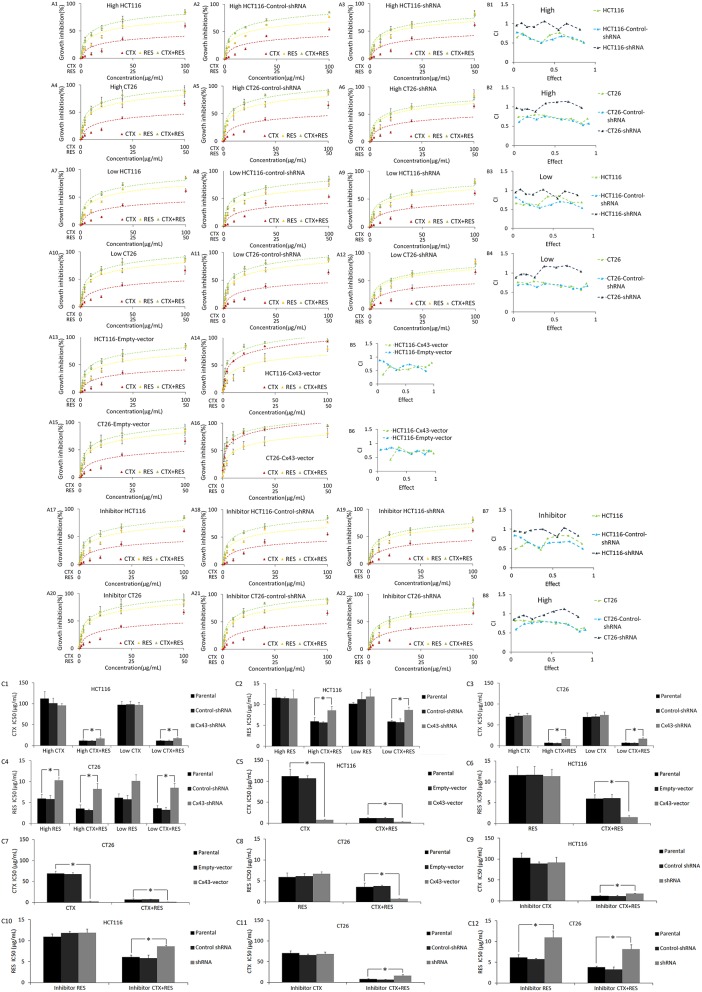
Results of the MTT assay and CalcuSyn software analysis for parental, shRNA transfected, and Cx43 transfected HCT116 and CT26 cells. vector, pTARGET vector; CTX, cetuximab treatment; RES, resveratrol treatment; High, high density cultured cells (80%); Low, low density cultured cells (5%). **(A)** MTT assay results. The vertical axis represents growth inhibition rate, which is compared with that of untreated cells. The horizontal axis represents agent concentrations. Points represent mean ± SEM. Panels (A13–A16) are the results of high density culture cells. The raw data of MTT are shown as Supplementary Document 1. **(B)** CalcuSyn software analysis of the MTT assay results. CI, combination index. CI is calculated by calcusyn analysis based on the inhibition rate of different concentration of resveratrol and cetuximab. CI > 1 represents antagonistic cytotoxicity; CI = 1 represents addictive cytotoxicity; CI < 1 represents synergistic cytotoxicity. The data generated by CalcuSyn are shown as Supplementary Document 1. **(C)** IC50 (μg/ml) of cells to resveratrol and/or cetuximab. *P < 0.05 represents a significant difference between parental and transfected cells compared by one-way ANOVA. There is no significant difference between parental and control-shRNA in all conditions. The statistical difference of growth inhibition between parental and Cx43-shRNA transfected cells in different concentration is shown as Supplementary Document 1.

The MTT assay showed that cetuximab and resveratrol both have considerable cytotoxicity in all four cell lines (Figure 2 and Figure S3). Cx43-shRNA transfection decreased the cytotoxicity of resveratrol in CT26, but not in HCT116. The relevant mechanism is still unknown. Cx43-shRNA transfection has no effect on the cytotoxicity of cetuximab. Furthermore, the combination of cetuximab and resveratrol has obvious synergy when administered to parental HCT116 or CT26 cells, but no clear synergy was observed in shRNA-transfected cells. Cx43-pTARGET vector transfection also increased the cytotoxicity of cetuximab in HCT116 and CT26 cells.

In addition, low density (5%), high density (80%), and carbenoxolone pretreated high density cultured cells are all measured by the MTT assay to determine whether this synergic effect depends on the GJ function. High density culture cells can form GJ among adjacent cells, which cannot be formed in low density culture. A GJ inhibitor, carbenoxolone, was also used to pretreat high density cultured cells to inhibit the GJ function. As shown in Figure S1C, carbenoxolone pretreatment significantly inhibited the GJ function in resveratrol treated parental cells. These results show that the GJ function does not contribute to this effect.

### Resveratrol Increases Cx43 Expression and GJ Function in Parental Cells

As shown in Figure 1 and Figure S4, almost all Cx43 expression is located in the cytoplasm rather than on the membrane of parental HCT116 cells. Parental CT26 cells have some Cx43 expression on the membrane, but the expression level is very low. Resveratrol increases Cx43 expression on the membrane effectively in these two parental cell lines. Phosphorylated Cx43 and the GJ function in these parental cells are also upregulated by resveratrol (Figure 1B); phosphorylated Cx43 in the membrane supports the GJ function (19). The GJ function of Cx43-shRNA-transfected cells is not affected by resveratrol treatment. Full-length blots of Figure 1A1 are shown in Figure S7A.

### Cx43 Interacts With Akt in Resveratrol-Treated Parental Cells

**Figure 3:**
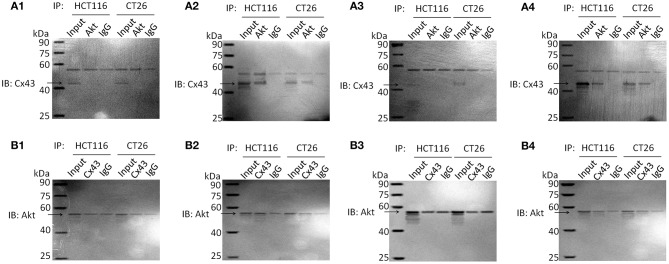
Immunoprecipitation analysis of Akt and Cx43 in parental HCT116 and CT26 cells. Cell lysates were subjected to immunoprecipitation with anti-Akt or anti-Cx43 antibodies. Then immunoprecipitated proteins were examined by western blotting with anti-Cx43 or anti-Akt antibodies, respectively. The input of cell total protein was used as a positive control and rabbit IgG was used as a negative control. Molecular weight of Akt and IgG heavy chain is similar, so they are hardly to be discriminated. SDS-PAGE of immunoprecipitation analysis is shown as Figure S5. **(A)** IB: Cx43; IP: Akt. **(A1)** Non-treated cells. **(A2)** Resveratrol-treated cells. **(A3)** Cetuximab-treated cells. **(A4)** Cetuximab + Resveratrol-treated cells. **(B)** IB: Akt; IP: Cx43. **(B1)** Non-treated cells. **(B2)** Resveratrol-treated cells. **(B3)** Cetuximab-treated cells. **(B4)** Cetuximab + Resveratrol-treated cells.

It has been reported that Cx43 interacts with Akt. In one study, proteasomal inhibition induced Akt activation, which resulted in the phosphorylation of Cx43 at Akt phosphorylation consensus sites in kidney cells (19). However, some reports have shown a negative correlation between Cx43 and Akt activation, caused by direct interactions between Cx43 and Akt in glioblastoma (15). In addition, activation of the PI3K-Akt pathway by 2-hydroxyflutamide resulted in reduced levels of Cx43 in rat Sertoli cells (18). Immunoprecipitation analysis (Figure 3 and Figure S5) showed an interaction between Cx43 and Akt in parental cells treated with resveratrol or with a combination of cetuximab and resveratrol. There is no obvious Cx43 band in non-treated parental cells or cetuximab-treated parental cells, perhaps because these cells may have Cx43 expression below the level of detection. Cx43-shRNA-transfected cells have almost no Cx43 expression, so they were not measured by immunoprecipitation assay. The results of immunocytochemistry analysis (Figure S4) also showed that resveratrol-treated and resveratrol- and cetuximab-treated parental cells have high Cx43 expression on the membrane, as well as considerable Akt expression in the same location.

### Resveratrol Inhibits Akt Pathway in Parental Cells

**Figure 4:**
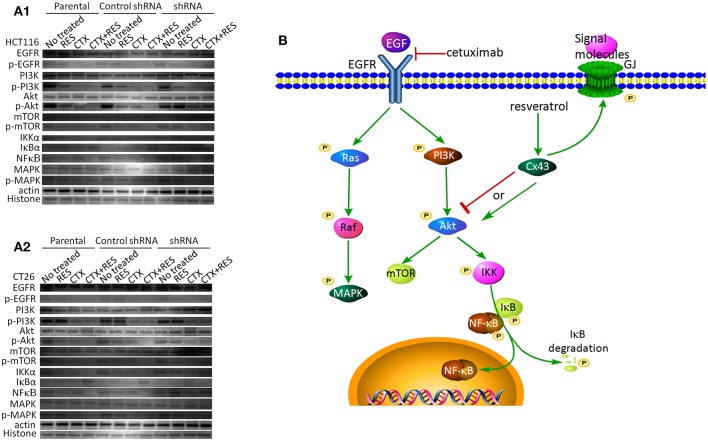
Western blot analysis of proteins related to the EGFR signal pathway, including EGFR, p-EGFR, PI3K, p-PI3K, Akt, p-Akt, mTOR, p-mTOR, IKKα, IκBα, NFκB p65, MAPK, and p-MAPK. **(A)** Western blot images. β-Actin is used as a loading control for total protein, and histone H3 is used as a loading control for nuclear proteins. CTX, cetuximab treatment; RES, resveratrol treatment. The full-length blots are shown as Figures S7C,D. **(B)** Relationship between the EGFR signaling pathway and Cx43 (1, 14, 15). Cetuximab inhibits the binding reaction of EGF to EGFR, which inhibits the whole EGFR signaling pathway. Akt, a key molecule in this pathway, interacts with Cx43. Resveratrol increases Cx43 expression and phosphorylation.

Cetuximab inhibits EGFR downstream signal pathways such as Ras-Raf-MAPK and PI3K-Akt via binding to EGFR (1). It has also been reported that resveratrol inhibits EGFR phosphorylation in ovarian cancer cells (12). Our results (Figure 4 and Figure S6) show that cetuximab inhibits these two signaling pathways in parental and shRNA-transfected HCT116 and CT26 cells, but resveratrol does not clearly inhibit EGFR, MAPK, and PI3K phosphorylation in parental and shRNA-transfected CT26 cells. Further experiments showed that resveratrol inhibits activation of Akt and downstream signaling pathways of Akt in parental cells, but not in shRNA-transfected cells. The combination resveratrol- and cetuximab-treated cells have the lowest activation level of Akt and downstream signaling pathways of Akt, including p-Akt, p-mTOR, IKKα, IκBα, and NFκB p65 in parental cells, but not in shRNA-transfected cells. So resveratrol may inhibit Akt and its downstream pathways by upregulating Cx43 (Figure 4B). Full-length blots of Figure 4A are shown in Figures S7C,D.

### “Resveratrol + Cetuximab” Treatment Has the Highest Inhibition of Tumorigenicity in Parental CT26 Cells *in vivo*

**Figure 5:**
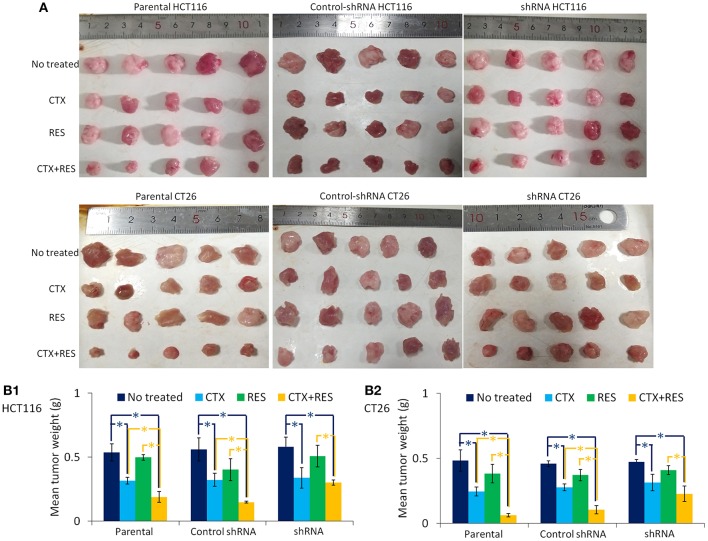
Inhibition of tumor growth by cetuximab and resveratrol. CTX, cetuximab-treated mice; RES, resveratrol-treated mice. **(A)** The subcutaneous tumor model was created by parental, control-shRNA, or Cx43-shRNA-transfected cells. Each group contains five Balb/c mice for CT26 or nu/nu nude mice for HCT116. These mice were then treated with cetuximab and/or resveratrol for 14 days before sacrificed. Tumors were removed, and weight was measured using electronic balance. **(B)** Columns represent mean ± SEM volume from five tumors. Groups “CTX,” “RES,” and “CTX+RES” were compared with group “No treated” using one-way ANOVA. Blue *P < 0.05 represents a significant difference. Group “CTX+RES” was compared with group “CTX” and “RES” using one-way ANOVA. Yellow *P < 0.05 represents a significant difference.

We found that the combination of resveratrol and cetuximab treatment has a significant inhibitory effect on tumor growth in the subcutaneous tumor model of parental CT26 and HCT116 cells but not of Cx43-shRNA-transfected cells (Figure 5). Briefly, cetuximab has a significant growth-inhibition effect on both parental and transfected cells that formed tumors. Resveratrol does not have significant tumorigenic inhibition effect on either parental or transfected cells, perhaps because it was used in low doses. Though Cx43-shRNA transfection decreased resveratrol cytotoxicity *in vitro* as shown in Figure 2, there is almost no difference in the tumor growth inhibition effect of resveratrol between parental and transfected cells *in vivo*. The combination treatment is effective in inhibiting tumor growth by parental cells in inoculated mice. Furthermore, Cx43-shRNA transfection did not affect tumor growth, which suggests that dislocation and low expression of Cx43 in parental cells cannot form effective GJ to control the growth of cells.

## Discussion

Cx43 is widely expressed in different human tissues and is a structural component of GJ, intercellular channels that transfer small water-soluble molecules and affect drug sensitivity and cancer metastasis (14). Cx43 also affects Akt activation through direct interaction (14); our results suggested that Cx43 interacts with Akt and inhibits its activation in colorectal cancer cells. We infer that this interaction may affect cetuximab sensitivity.

In general, connexins form GJ, inducing contact inhibition of cell growth; hence, it behaves like a tumor suppressor in the initial stages of cancer. In colorectal cancer, Cx43 is downregulated or is expressed in the wrong location and cannot form effective GJ (24). Cx43 forms GJ effectively only when expressed on the cell membrane and only when phosphorylated (19). Cx43 has different effects on different cancer types. It has a protective effect against angiogenesis in breast cancer cells (25), but significantly increases tubulogenesis when glioma cells are co-cultured with human umbilical vascular endothelial cells (26). Some studies indicate that resveratrol increases Cx43 expression and GJ in cancer cells such as those of hepatocellular carcinoma (27) and melanoma (28). Akt is one of the resveratrol-associated predicted targets on colorectal cancer (29) and is regulated by Cx43 in many cases of antitumor studies. For example, the C-terminus of Cx43 recruits Src and PTEN to inhibit Akt activation in glioma cells (30). Disruption of Cx43 also enhanced the phosphorylation of Src and Akt (31). Our results showed that resveratrol increased Cx43 phosphorylation and expression on the membranes of parental cells, so that resveratrol upregulated the GJ function of parental cells. We suggest that this effect may cause Akt inhibition through the interaction between Cx43 and Akt, because there is no obvious Akt inhibition in resveratrol-treated Cx43-shRNA transfected cells. We confirmed that the sensitivity of colorectal cancer cells to cetuximab was significantly increased by resveratrol-induced Akt inhibition.

As an EGFR antibody, cetuximab has a higher binding affinity (*K*_d_ = 0.1–0.2 nM) to EGFR than endogenous ligands do. This binding reaction inhibits the downstream signaling pathways of receptor tyrosine kinase, including Ras-Raf-MAPK and PI3K-Akt (1). Oncogenic activation of these two pathways is a critical mechanism to generate cetuximab resistance. For example, phosphoinositide 3-kinase catalytic subunit α has activating mutations in some metastatic colorectal cancer tissues, which are used to predict cetuximab resistance (32). The status of Akt activation also relates to anti-EGFR therapy resistance in head and neck squamous cell carcinoma (33) and lung cancer (2). Hence, the PI3K-Akt pathway cannot be totally inhibited by cetuximab. Therefore, cetuximab needs to be combined with other drugs to solve this problem. Besides inhibition of receptor tyrosine kinase, many factors cause Akt inactivation, such as protein phosphatase A2, PH domain leucine-rich repeat protein phosphatase, or PTEN hyperactivity (34). Our results show that Cx43 also inhibits Akt activation in colorectal cancer cells; therefore, agents that increase Cx43 expression may sensitize colorectal cancer cells to cetuximab. By comparing the IC50 of “high density,” “low density,” and “carbenoxolone pretreated” groups, we determined that the GJ function is not required to sensitize colorectal cancer cells to cetuximab. It is also implied that the GJ function may not be required for Akt inhibition induced by resveratrol.

The ability of resveratrol to inhibit Akt has been used in many combined therapy strategies with resveratrol and other antitumor agents. For example, resveratrol suppresses NF-κB activity via Akt inhibition to enhance TRAIL cytotoxicity in lung cancer (5). It is also reported that PI3K is inhibited by resveratrol in prostate cancer, which causes PI3K-Akt inhibition and modulations in Bcl-2 family to promote apoptosis (35). However, there are contradictory reports stating that resveratrol was found to activate PI3K-Akt in paclitaxel-treated cells (7). Our results do not show that resveratrol inhibits PI3K in HCT116 and CT26 cells; thus, resveratrol may inhibit Akt mainly by upregulating Cx43 in these cells. A downstream protein of the PI3K-Akt pathway, NF-κB, was also measured to evaluate the effect of resveratrol and cetuximab. NF-κB is a pro-inflammatory transcription factor activated by carcinogens, tumor promoters, and some antitumor agents. The PI3K-Akt pathway plays a key role in the regulation of apoptosis by NF-κB (36). NF-κB activation results in resistance to many chemotherapeutic drugs, whereas its suppression is known to provide effective therapeutic benefits in chemotherapy treatments (20, 21). We found that resveratrol and cetuximab both inhibit NF-κB in these cells, and cells treated with the combination of these two agents have the lowest NF-κB level in their nuclei. These results suggest that the combination of cetuximab and resveratrol has an ideal synergistic effect.

In summary, as an anti-EGFR agent, cetuximab blocks downstream signal transduction pathways of EGF/EGFR. Akt activation is an important factor of cetuximab resistance. Akt activation can be affected by Cx43. It has been reported that resveratrol can regulate Akt and increase Cx43, but resveratrol and Cx43 have different effects on Akt in different tissue types. In our study, we confirmed that resveratrol inhibits Akt and NF-κB by upregulating Cx43 in colorectal cancer cell lines HCT116 and CT26. Resveratrol was found to sensitize colorectal cancer cells to cetuximab *in vitro* and *in vivo*. This study also suggests that Cx43 is a target for the development of new therapeutic strategies to overcome cetuximab resistance.

## Data Availability Statement

All datasets generated for this study are included in the article/Supplementary Material.

## Ethics Statement

The animal study was reviewed and approved by Ethics committee of Tianjin Union Medical Center.

## Author Contributions

YW and WW drafted the manuscript, performed Cx43 knockdown, drug treatment, western blots, immunoprecipitation, animal experiments, and MTT assay. CL performed “Parachute” dye-coupling assay. YH performed immunocytochemistry experiments. XW and HZ performed Cx43 transfection and western blots experiments in the process of manuscript revision. YC designed and supervised the study.

### Conflict of Interest

The authors declare that the research was conducted in the absence of any commercial or financial relationships that could be construed as a potential conflict of interest.
