# Supplementary material for: Resveratrol Sensitizes Colorectal Cancer Cells to Cetuximab by Connexin 43 Upregulation-Induced Akt Inhibition
Source: Front Oncol. 2020 Apr 7;10:383. doi: 10.3389/fonc.2020.00383 (PMC7155766; doi:10.3389/fonc.2020.00383)
Supplement: Supplementary file 1 [file Data_Sheet_1.pdf]

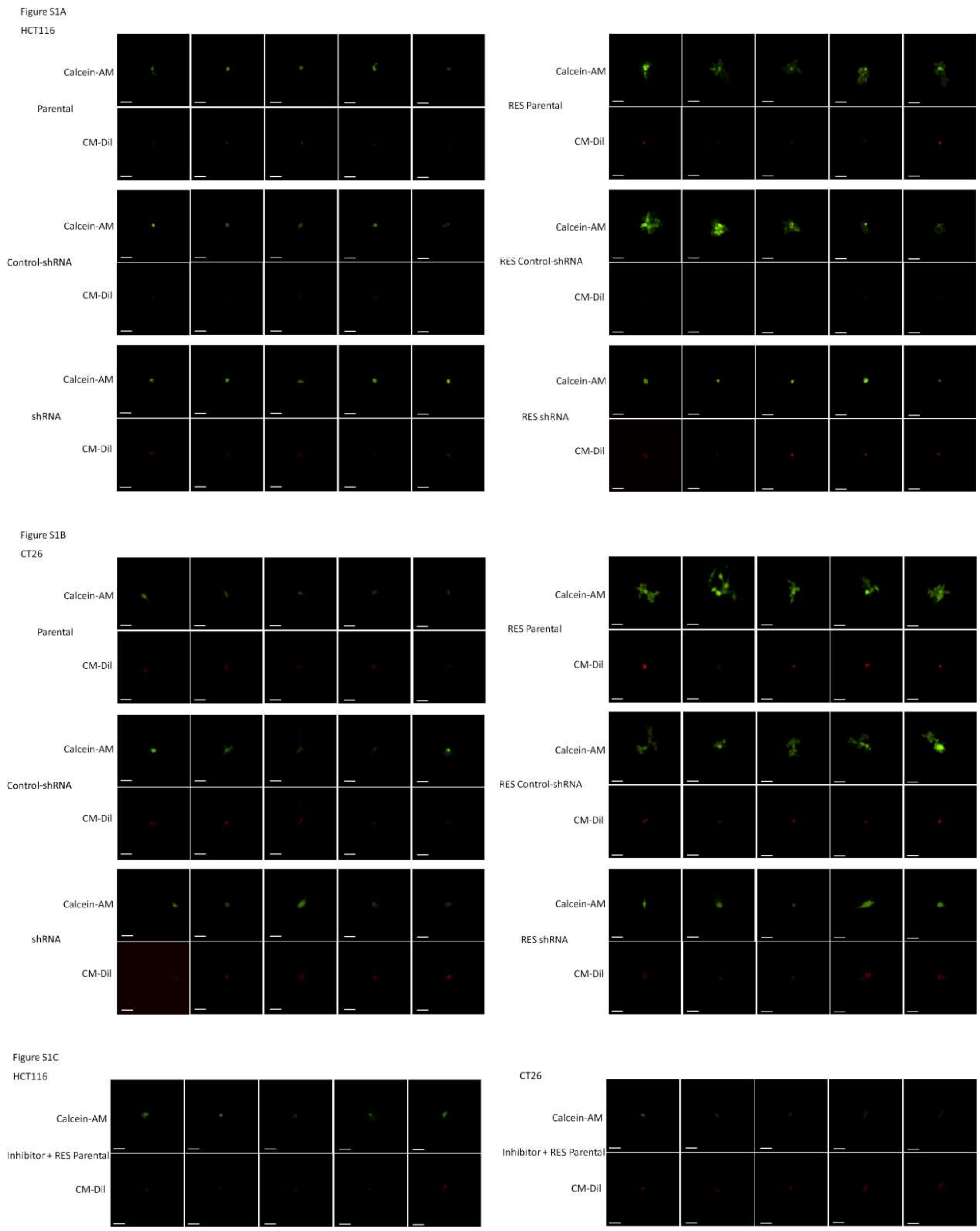

Figure S1. Five independent experiments of "Parachute" dye-coupling assay. Scale bars are 20 μm. (A, B) Five independent experiments of Figure 1B. (C) "Parachute" dye-coupling assay of cells which were pretreated with 50μM carbenoxolone for 12 h and treated with 5μg/ml resveratrol for 24 h. "Inhibitor" represents carbenoxolone. It is a significant difference (P<0.05) in GJ function between "Inhibitor+RES Parental" group of and "RES Parental" group by one-way ANOVA comparison.

Figure S2

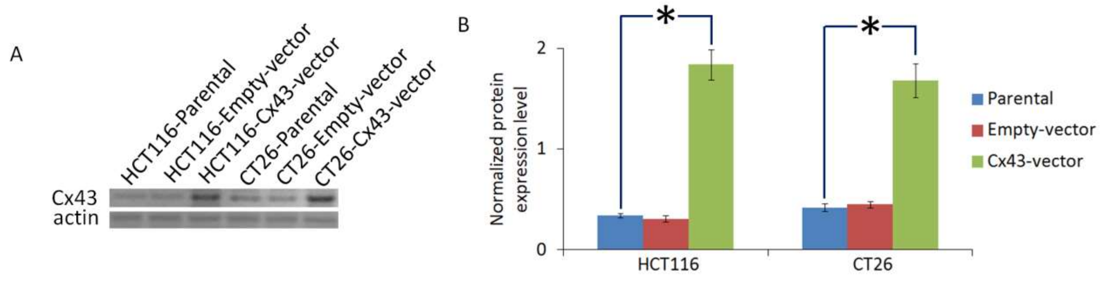

Figure S2. Cx43 expression is upregulated by transfection of Cx43-pTARGET vector. (A) Western blot images of Cx43 expression in empty-vector or Cx43-vector transfected HCT116 and CT26 cells. The full length blots are shown as Figure S7B. (B) Bar diagrams of densitometric analysis. Protein expression level is normalized by β-actin. Columns show the mean± SEM. All other groups were compared with the Parental group using one-way ANOVA. *P<0.05 represents a significant difference from values in the Parental group.

Figure S3A

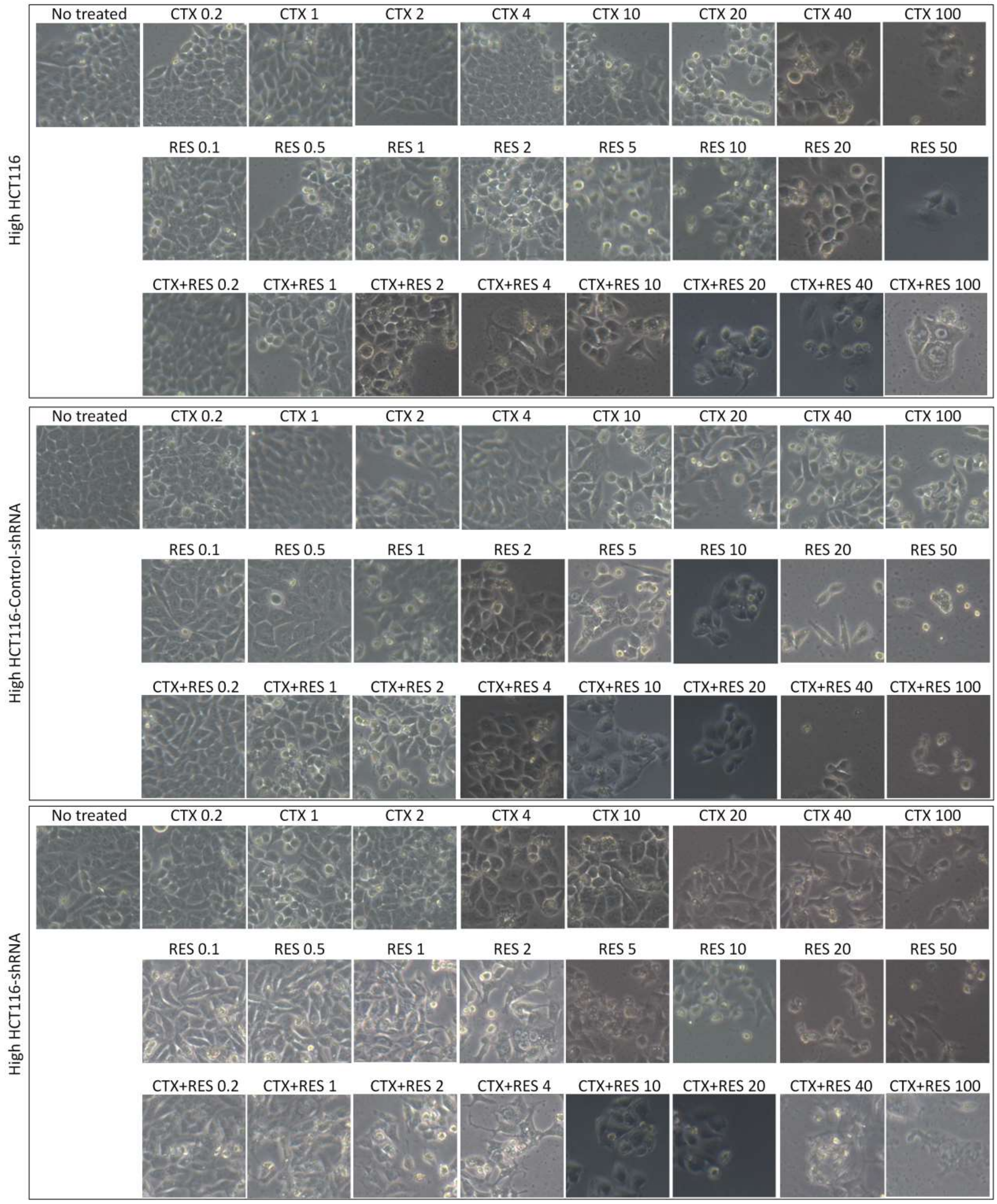
High HCT116
No treated
CTX 0.2
CTX 1
CTX 2
CTX 4
CTX 10
CTX 20
CTX 40
CTX 100
RES 0.1
RES 0.5
RES 1
RES 2
RES 5
RES 10
RES 20
RES 50
CTX+RES 0.2
CTX+RES 1
CTX+RES 2
CTX+RES 4
CTX+RES 10
CTX+RES 20
CTX+RES 40
CTX+RES 100
High HCT116-Control-shRNA
No treated
CTX 0.2
CTX 1
CTX 2
CTX 4
CTX 10
CTX 20
CTX 40
CTX 100
RES 0.1
RES 0.5
RES 1
RES 2
RES 5
RES 10
RES 20
RES 50
CTX+RES 0.2
CTX+RES 1
CTX+RES 2
CTX+RES 4
CTX+RES 10
CTX+RES 20
CTX+RES 40
CTX+RES 100
High HCT116-shRNA
No treated
CTX 0.2
CTX 1
CTX 2
CTX 4
CTX 10
CTX 20
CTX 40
CTX 100
RES 0.1
RES 0.5
RES 1
RES 2
RES 5
RES 10
RES 20
RES 50
CTX+RES 0.2
CTX+RES 1
CTX+RES 2
CTX+RES 4
CTX+RES 10
CTX+RES 20
CTX+RES 40
CTX+RES 100

Figure S3B

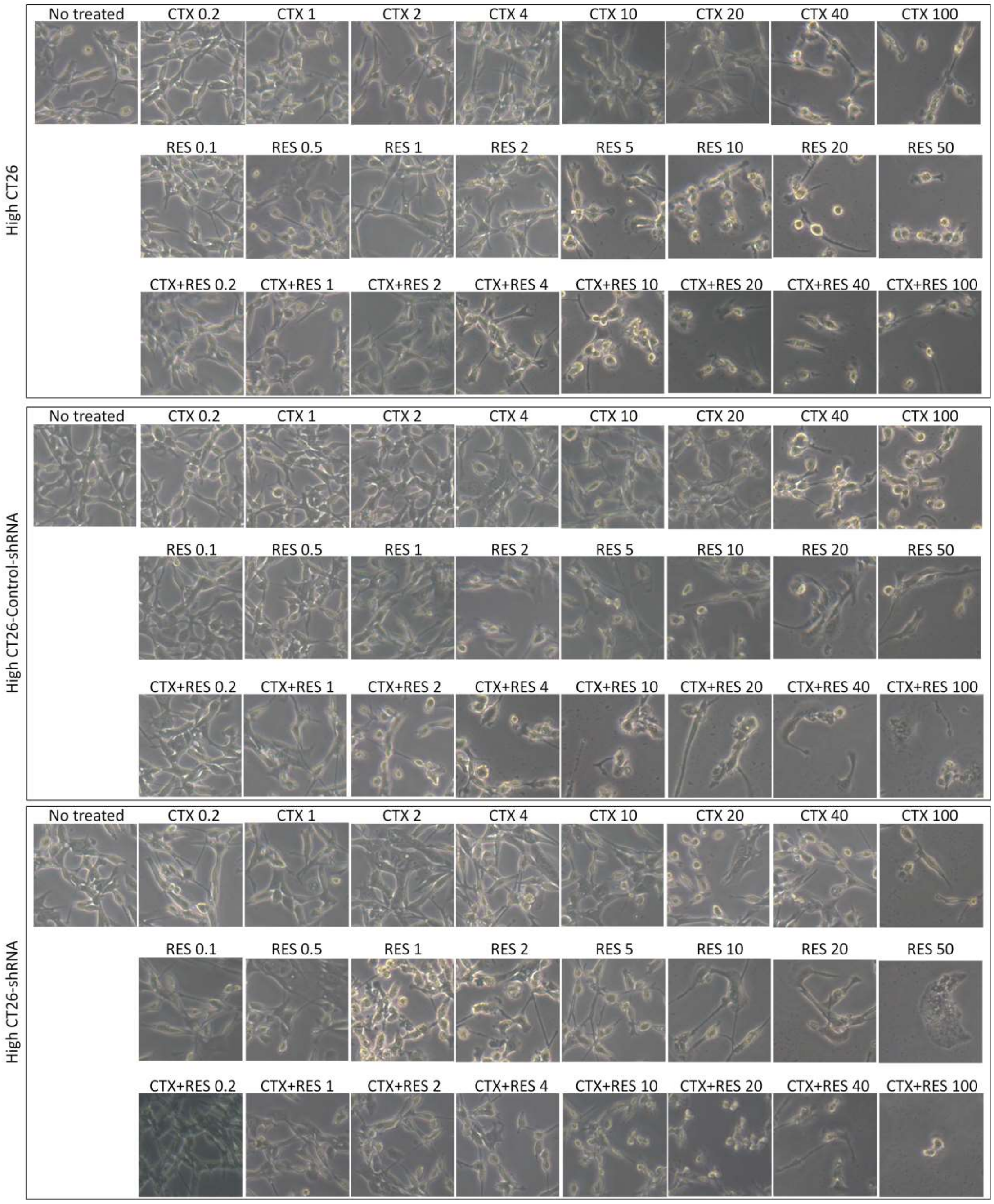
High CT26
No treated
CTX 0.2
CTX 1
CTX 2
CTX 4
CTX 10
CTX 20
CTX 40
CTX 100
RES 0.1
RES 0.5
RES 1
RES 2
RES 5
RES 10
RES 20
RES 50
CTX+RES 0.2
CTX+RES 1
CTX+RES 2
CTX+RES 4
CTX+RES 10
CTX+RES 20
CTX+RES 40
CTX+RES 100
High CT26-Control-shRNA
No treated
CTX 0.2
CTX 1
CTX 2
CTX 4
CTX 10
CTX 20
CTX 40
CTX 100
RES 0.1
RES 0.5
RES 1
RES 2
RES 5
RES 10
RES 20
RES 50
CTX+RES 0.2
CTX+RES 1
CTX+RES 2
CTX+RES 4
CTX+RES 10
CTX+RES 20
CTX+RES 40
CTX+RES 100
High CT26-shRNA
No treated
CTX 0.2
CTX 1
CTX 2
CTX 4
CTX 10
CTX 20
CTX 40
CTX 100
RES 0.1
RES 0.5
RES 1
RES 2
RES 5
RES 10
RES 20
RES 50
CTX+RES 0.2
CTX+RES 1
CTX+RES 2
CTX+RES 4
CTX+RES 10
CTX+RES 20
CTX+RES 40
CTX+RES 100

Figure S3C

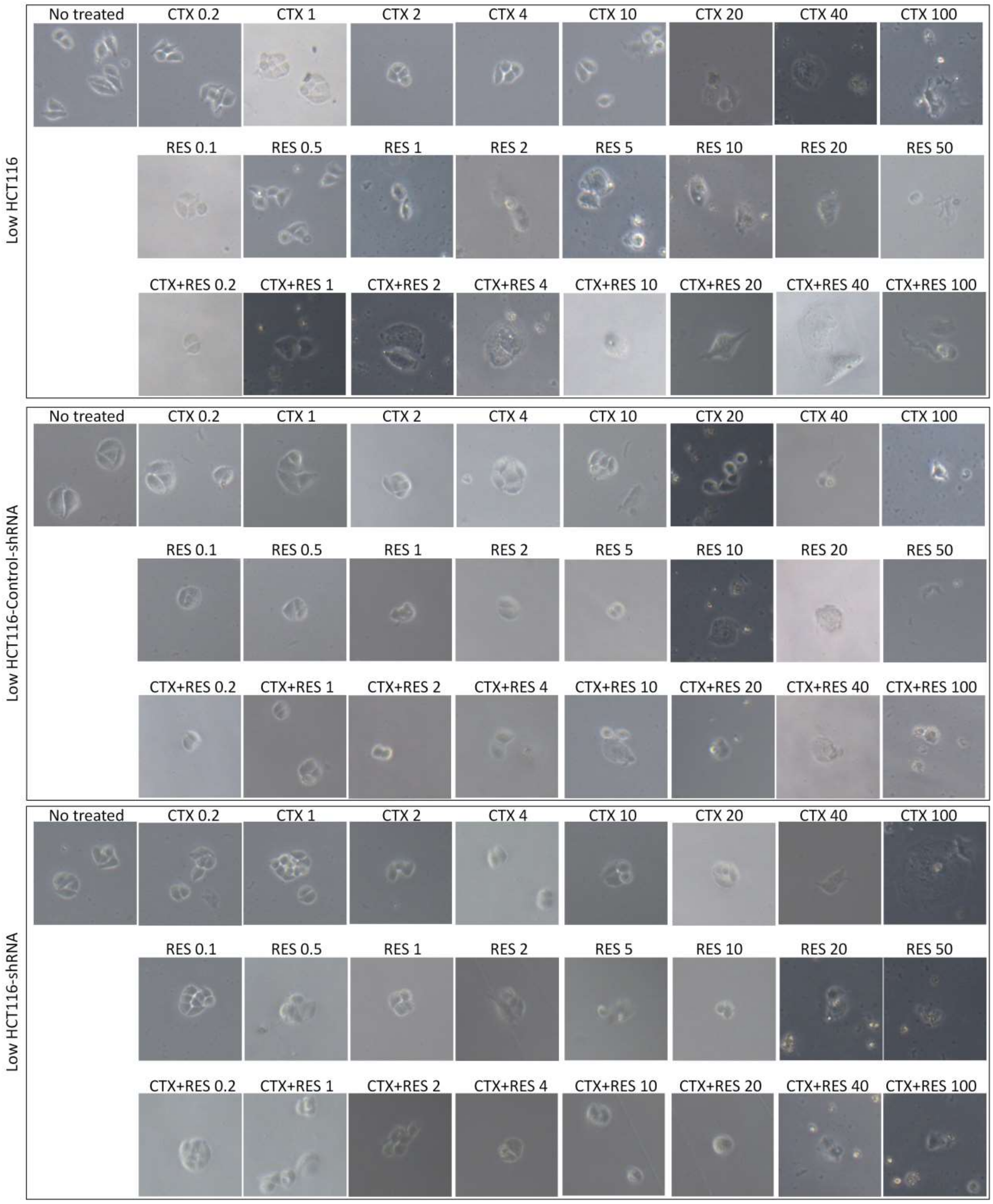
Low HCT116
Low HCT116-Control-shRNA
Low HCT116-shRNA
No treated
CTX 0.2
CTX 1
CTX 2
CTX 4
CTX 10
CTX 20
CTX 40
CTX 100
RES 0.1
RES 0.5
RES 1
RES 2
RES 5
RES 10
RES 20
RES 50
CTX+RES 0.2
CTX+RES 1
CTX+RES 2
CTX+RES 4
CTX+RES 10
CTX+RES 20
CTX+RES 40
CTX+RES 100

Figure S3D

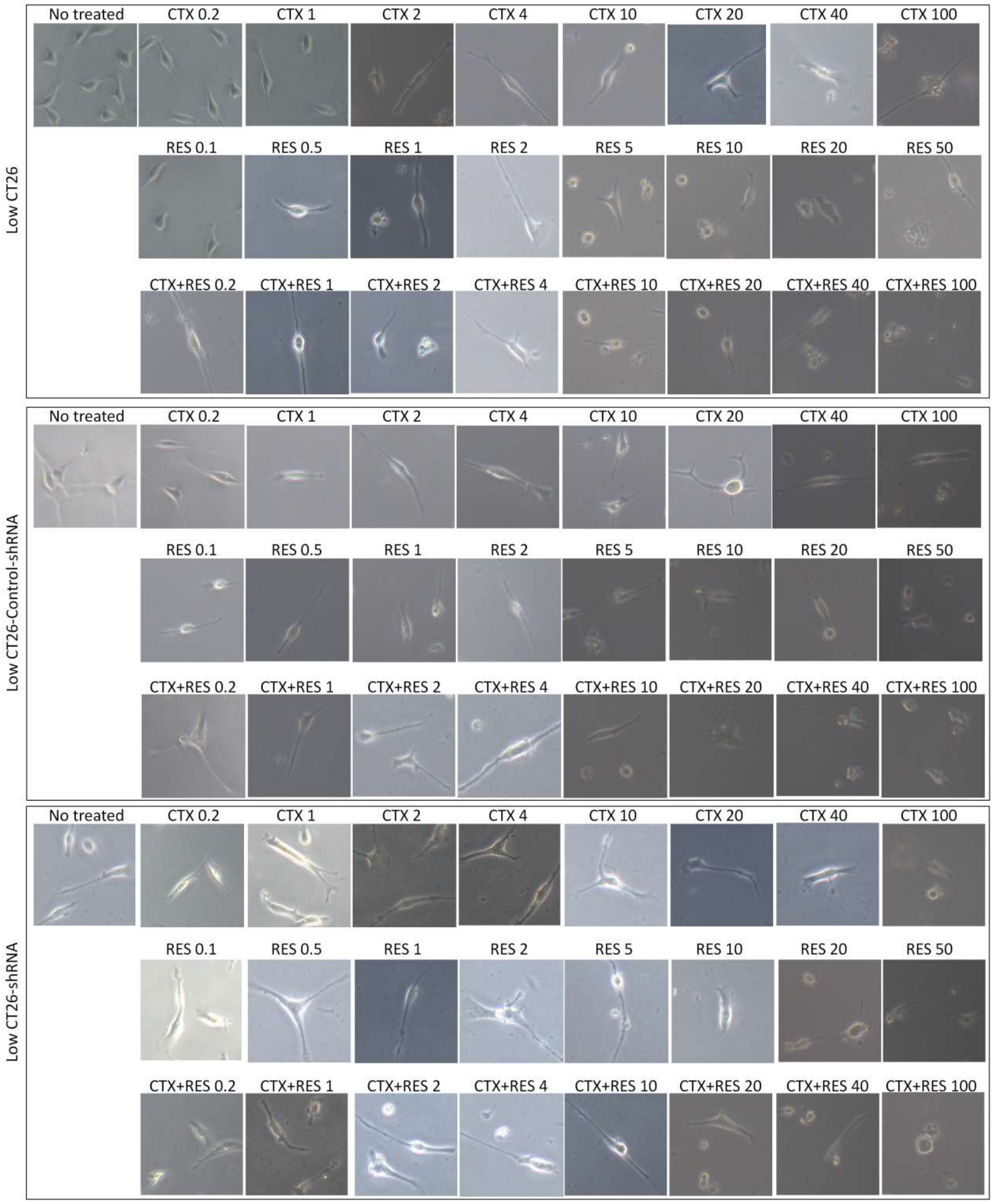

Low CT26
No treated
CTX 0.2
CTX 1
CTX 2
CTX 4
CTX 10
CTX 20
CTX 40
CTX 100
RES 0.1
RES 0.5
RES 1
RES 2
RES 5
RES 10
RES 20
RES 50
CTX+RES 0.2
CTX+RES 1
CTX+RES 2
CTX+RES 4
CTX+RES 10
CTX+RES 20
CTX+RES 40
CTX+RES 100
Low CT26-Control-shRNA
No treated
CTX 0.2
CTX 1
CTX 2
CTX 4
CTX 10
CTX 20
CTX 40
CTX 100
RES 0.1
RES 0.5
RES 1
RES 2
RES 5
RES 10
RES 20
RES 50
CTX+RES 0.2
CTX+RES 1
CTX+RES 2
CTX+RES 4
CTX+RES 10
CTX+RES 20
CTX+RES 40
CTX+RES 100
Low CT26-shRNA
No treated
CTX 0.2
CTX 1
CTX 2
CTX 4
CTX 10
CTX 20
CTX 40
CTX 100
RES 0.1
RES 0.5
RES 1
RES 2
RES 5
RES 10
RES 20
RES 50
CTX+RES 0.2
CTX+RES 1
CTX+RES 2
CTX+RES 4
CTX+RES 10
CTX+RES 20
CTX+RES 40
CTX+RES 100

Figure S3E

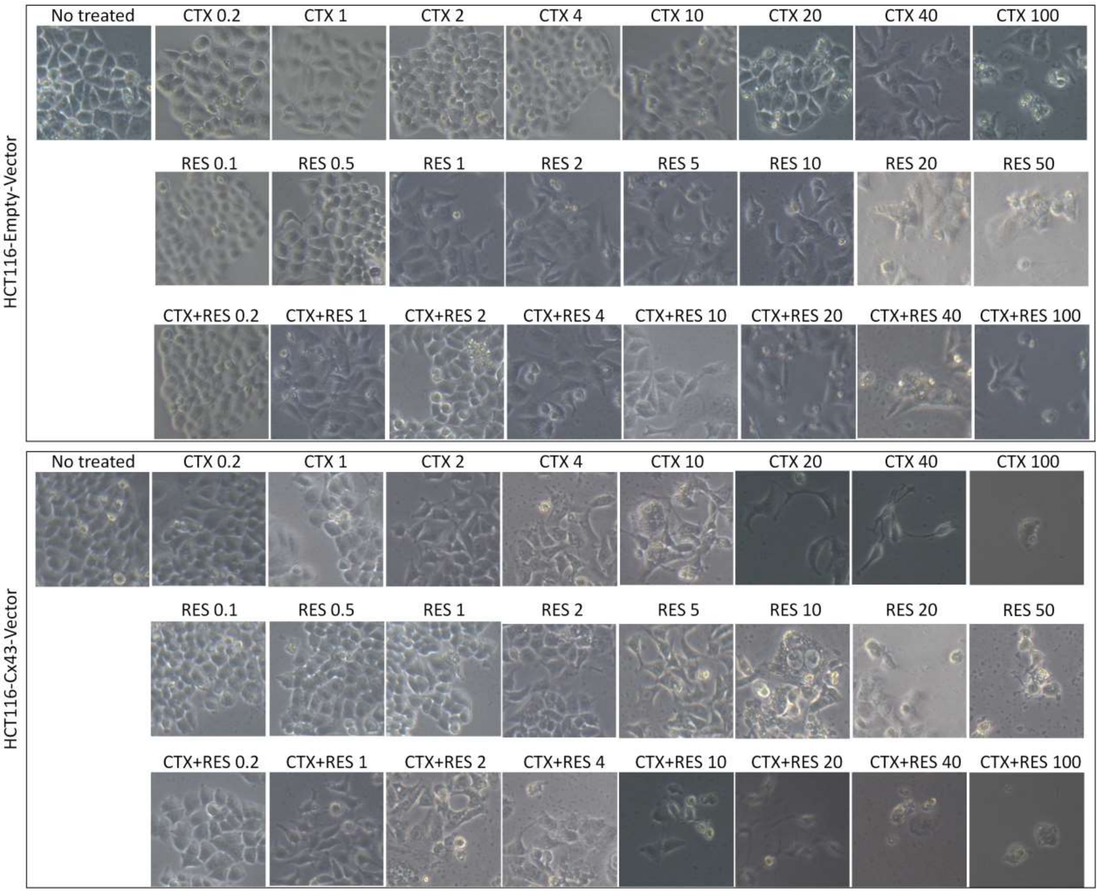
HCT116-Empty-Vector
No treated
CTX 0.2
CTX 1
CTX 2
CTX 4
CTX 10
CTX 20
CTX 40
CTX 100
RES 0.1
RES 0.5
RES 1
RES 2
RES 5
RES 10
RES 20
RES 50
CTX+RES 0.2
CTX+RES 1
CTX+RES 2
CTX+RES 4
CTX+RES 10
CTX+RES 20
CTX+RES 40
CTX+RES 100
HCT116-Cx43-Vector
No treated
CTX 0.2
CTX 1
CTX 2
CTX 4
CTX 10
CTX 20
CTX 40
CTX 100
RES 0.1
RES 0.5
RES 1
RES 2
RES 5
RES 10
RES 20
RES 50
CTX+RES 0.2
CTX+RES 1
CTX+RES 2
CTX+RES 4
CTX+RES 10
CTX+RES 20
CTX+RES 40
CTX+RES 100

Figure S3F

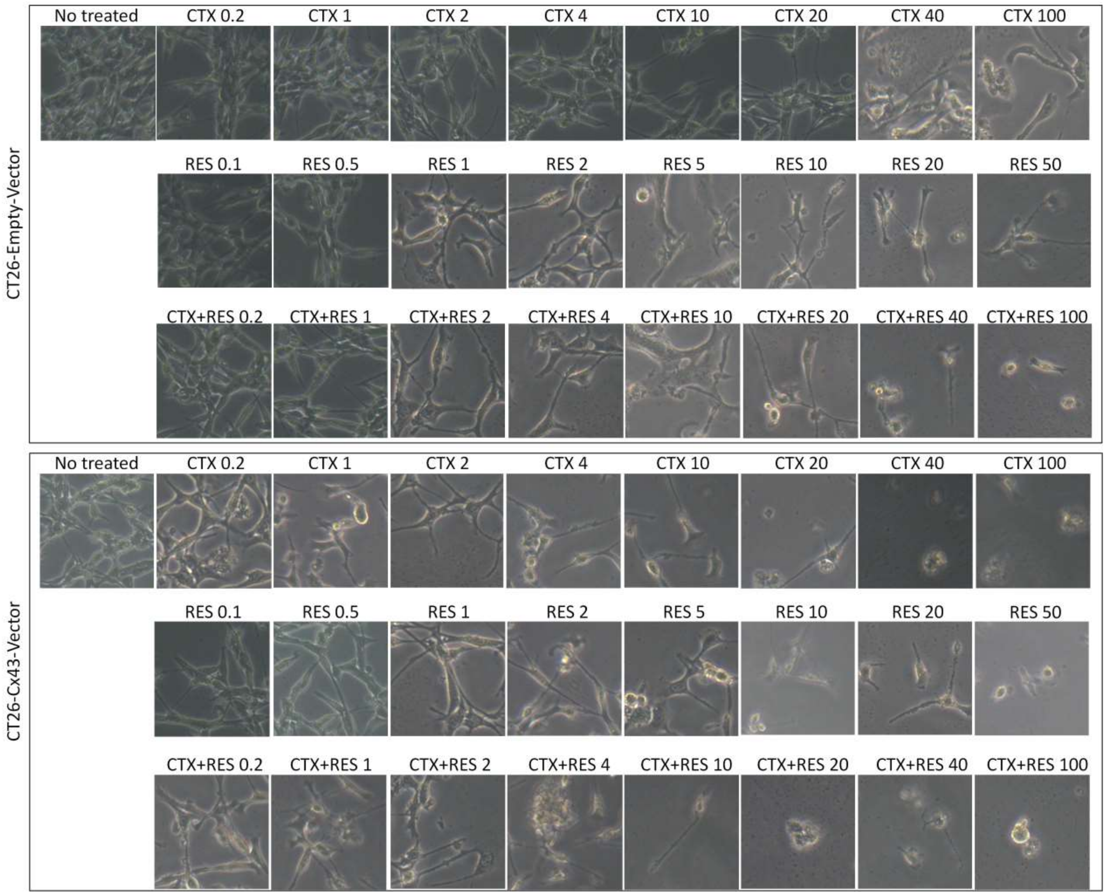
CT26-Empty-Vector
No treated
CTX 0.2
CTX 1
CTX 2
CTX 4
CTX 10
CTX 20
CTX 40
CTX 100
RES 0.1
RES 0.5
RES 1
RES 2
RES 5
RES 10
RES 20
RES 50
CTX+RES 0.2
CTX+RES 1
CTX+RES 2
CTX+RES 4
CTX+RES 10
CTX+RES 20
CTX+RES 40
CTX+RES 100
CT26-Cx43-Vector
No treated
CTX 0.2
CTX 1
CTX 2
CTX 4
CTX 10
CTX 20
CTX 40
CTX 100
RES 0.1
RES 0.5
RES 1
RES 2
RES 5
RES 10
RES 20
RES 50
CTX+RES 0.2
CTX+RES 1
CTX+RES 2
CTX+RES 4
CTX+RES 10
CTX+RES 20
CTX+RES 40
CTX+RES 100

Figure S3G

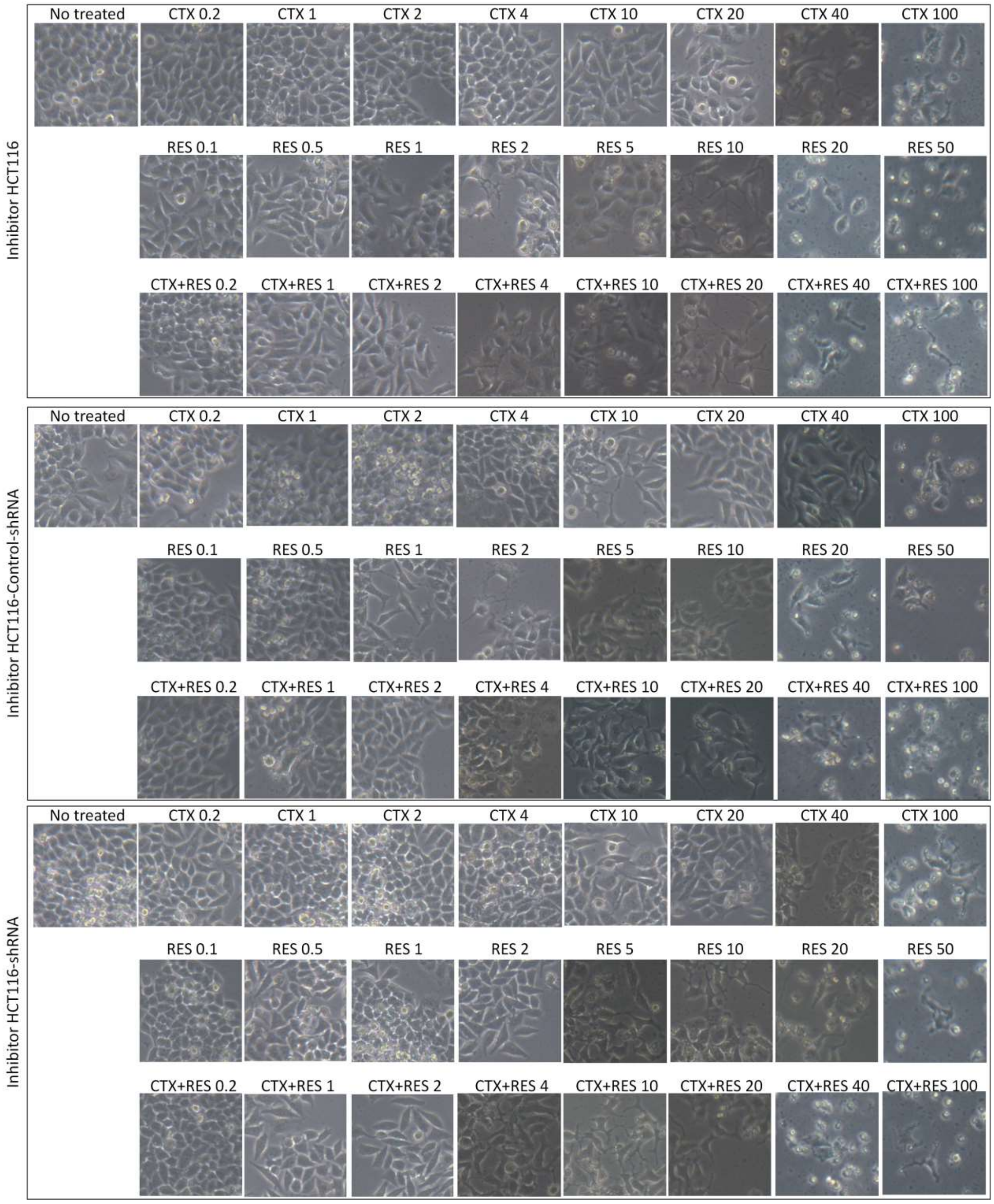
Inhibitor HCT116
No treated
CTX 0.2
CTX 1
CTX 2
CTX 4
CTX 10
CTX 20
CTX 40
CTX 100
RES 0.1
RES 0.5
RES 1
RES 2
RES 5
RES 10
RES 20
RES 50
CTX+RES 0.2
CTX+RES 1
CTX+RES 2
CTX+RES 4
CTX+RES 10
CTX+RES 20
CTX+RES 40
CTX+RES 100
Inhibitor HCT116-Control-shRNA
No treated
CTX 0.2
CTX 1
CTX 2
CTX 4
CTX 10
CTX 20
CTX 40
CTX 100
RES 0.1
RES 0.5
RES 1
RES 2
RES 5
RES 10
RES 20
RES 50
CTX+RES 0.2
CTX+RES 1
CTX+RES 2
CTX+RES 4
CTX+RES 10
CTX+RES 20
CTX+RES 40
CTX+RES 100
Inhibitor HCT116-shRNA
No treated
CTX 0.2
CTX 1
CTX 2
CTX 4
CTX 10
CTX 20
CTX 40
CTX 100
RES 0.1
RES 0.5
RES 1
RES 2
RES 5
RES 10
RES 20
RES 50
CTX+RES 0.2
CTX+RES 1
CTX+RES 2
CTX+RES 4
CTX+RES 10
CTX+RES 20
CTX+RES 40
CTX+RES 100

Figure S3H

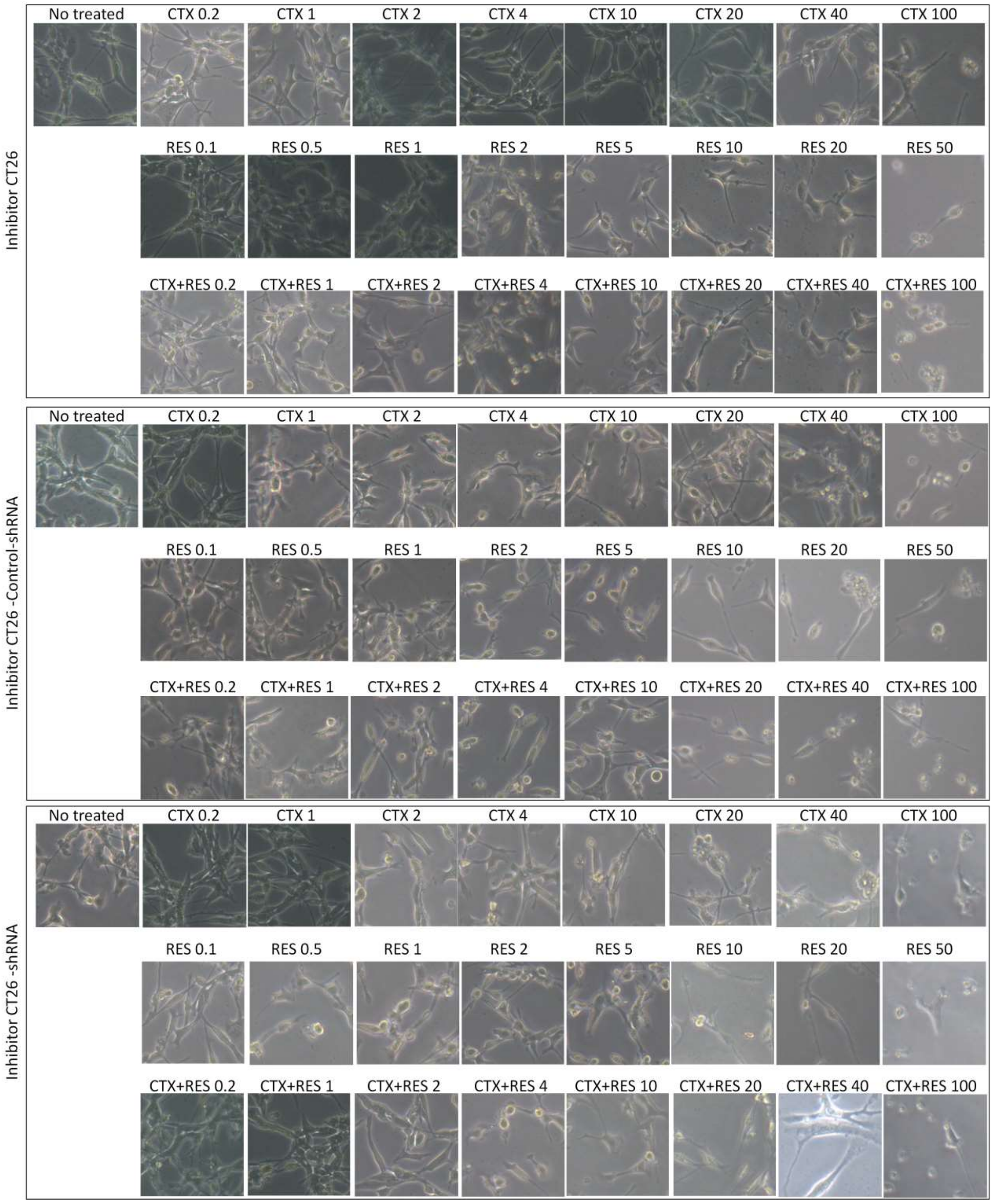

Figure S3. Photomicrographs of the cells under different treatments. "CTX" represents cetuximab treatment, "RES" represents resveratrol treatment, "CTX+RES" represents combination treatment. The number represents concentration (μg/mL) of drug, and the number of "CTX+RES" represents concentration of cetuximab. "Inhibitor" represents carbenoxolone pretreatment.

Figure S4A

Parental HCT116

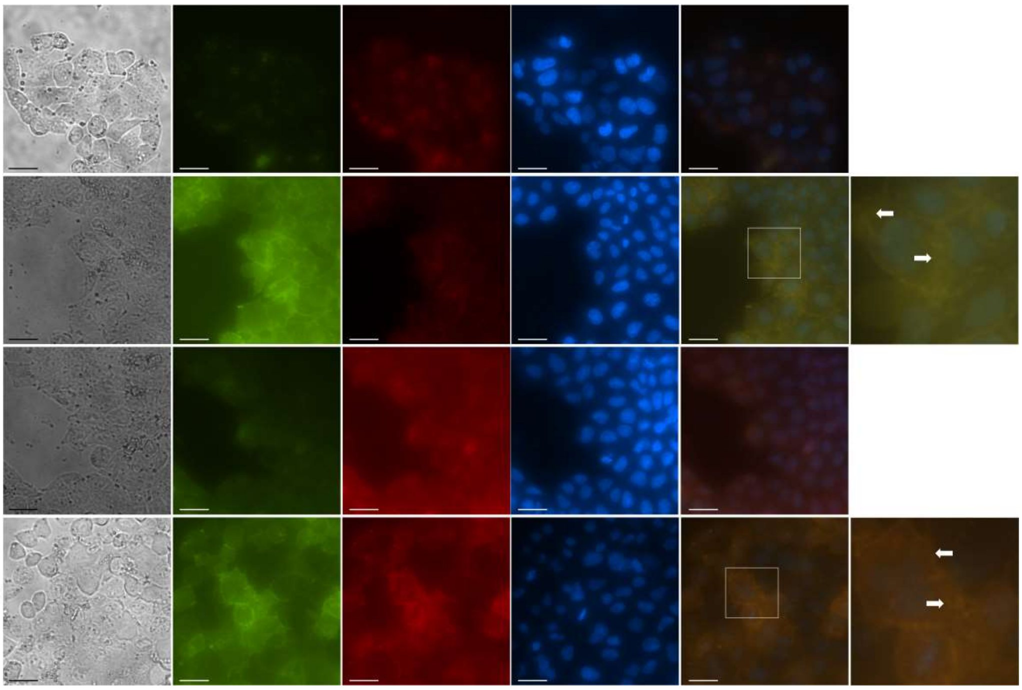

Figure S4B

Control-shRNA HCT116

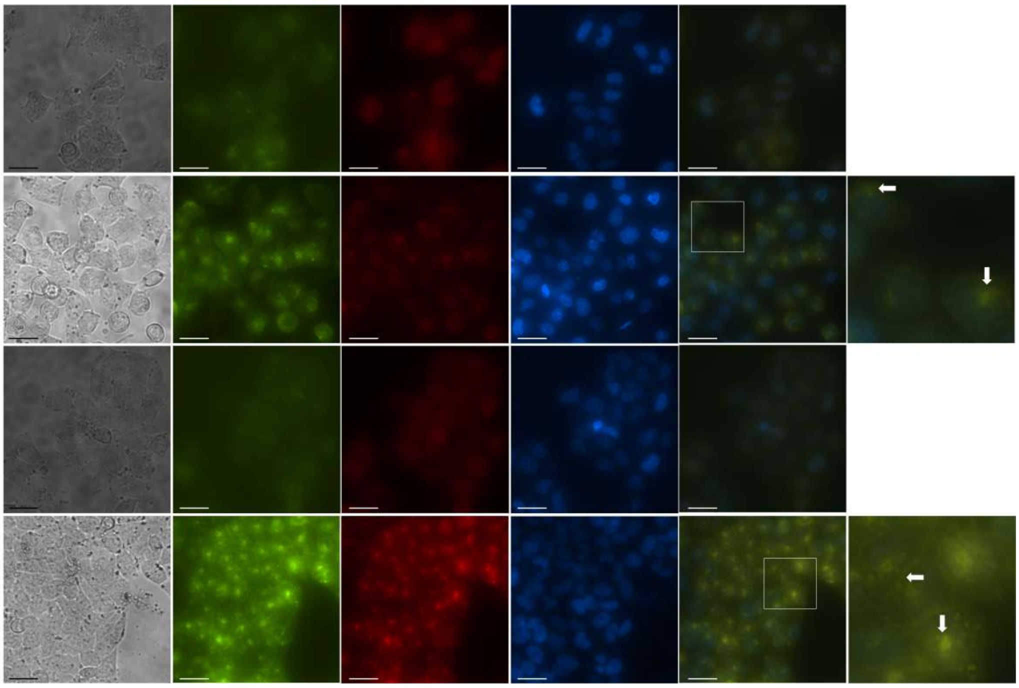

Figure S4C

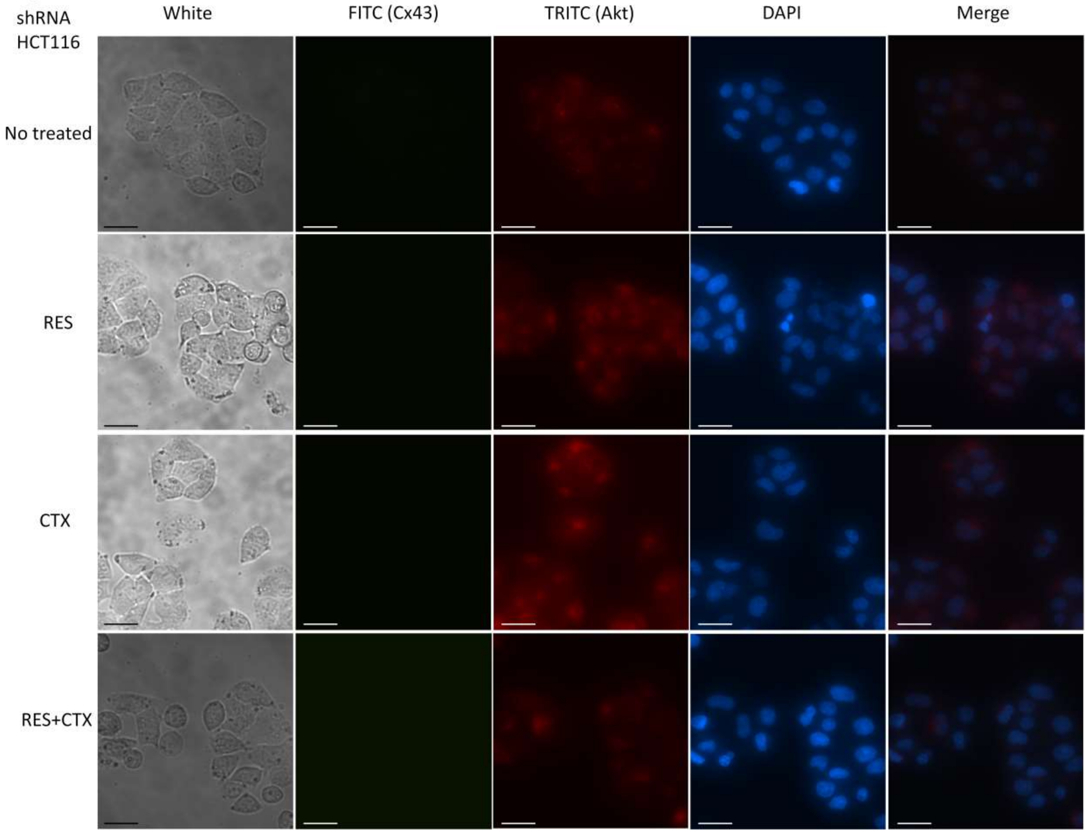

Figure S4D

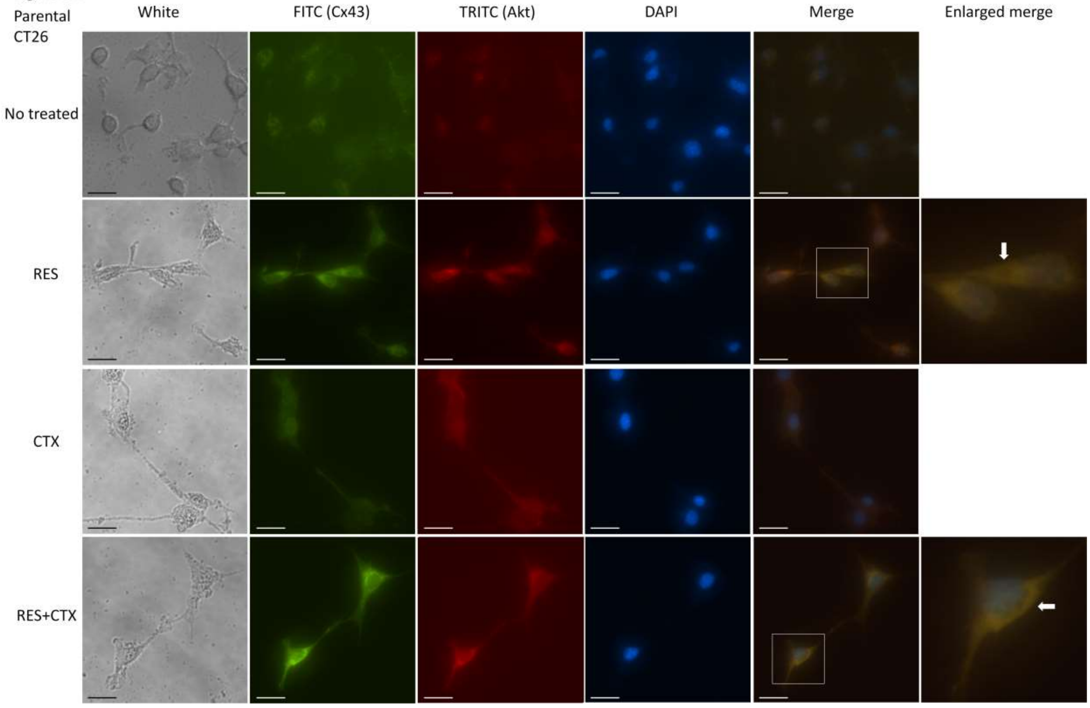

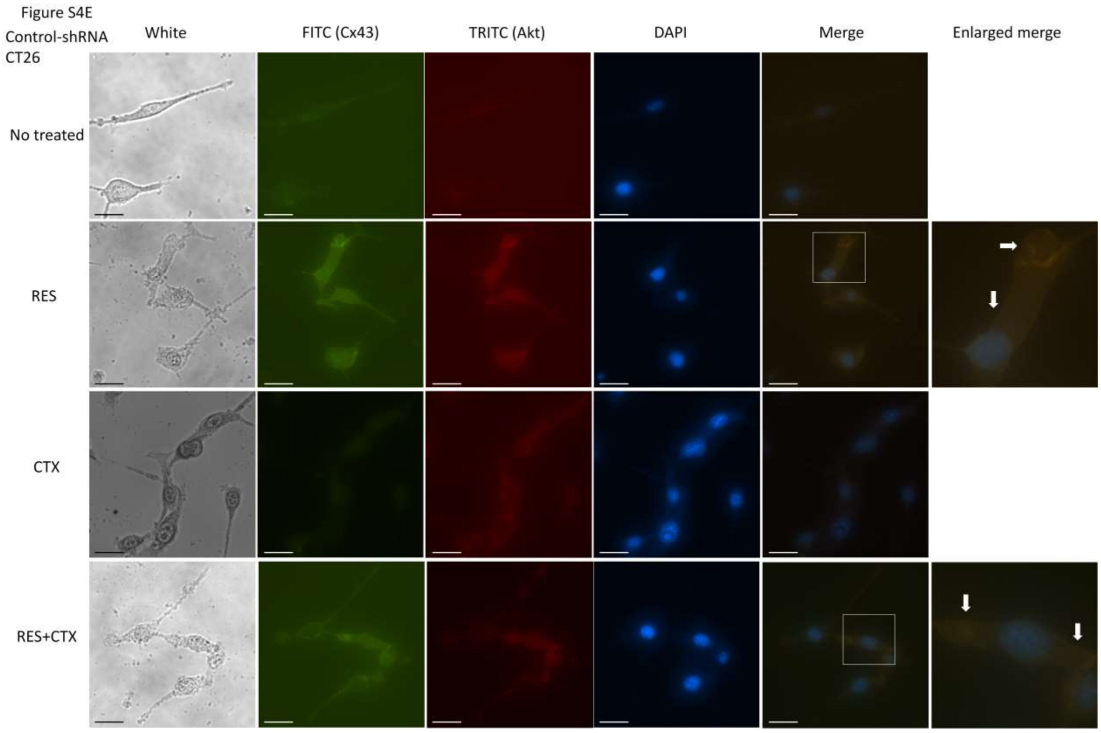

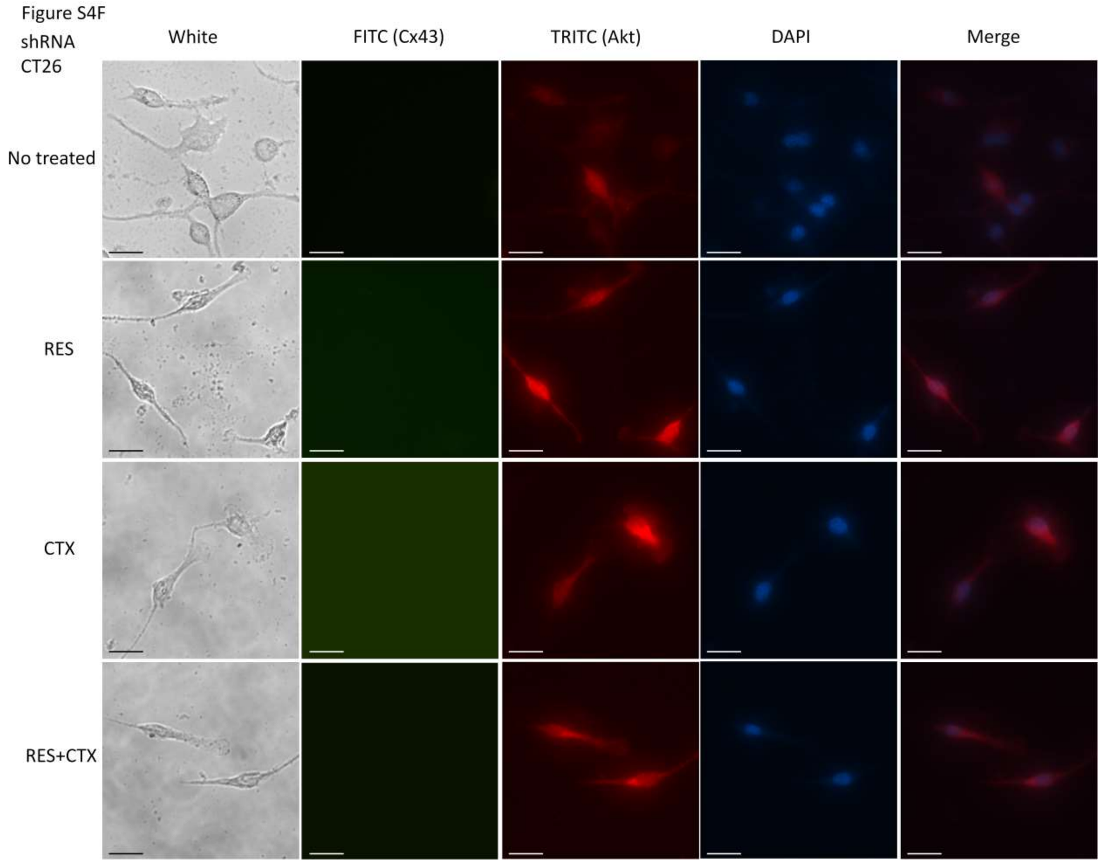

Figure S4. The location of Cx43 and Akt expression in parental and Cx43-shRNA-transfected cells. "RES" represents resveratrol-treated cells, "CTX" represents cetuximab-treated cells, "RES+CTX" represents the combination of resveratrol- and

cetuximab-treated cells. Cells were immunostained with Cx43 and Akt antibodies, which were followed by FITC- and TRITC-conjugated antibodies. Some area is enlarged to show colocalization of Akt and Cx43. Scale bars are 20 μm. (A) Parental HCT116 cells. (B) Control-shRNA transfected HCT116 cells. (C) shRNA transfected HCT116 cells. (D) Parental CT26 cells. (E) Control-shRNA transfected CT26 cells. (F) shRNA transfected CT26 cells.

Figure S5

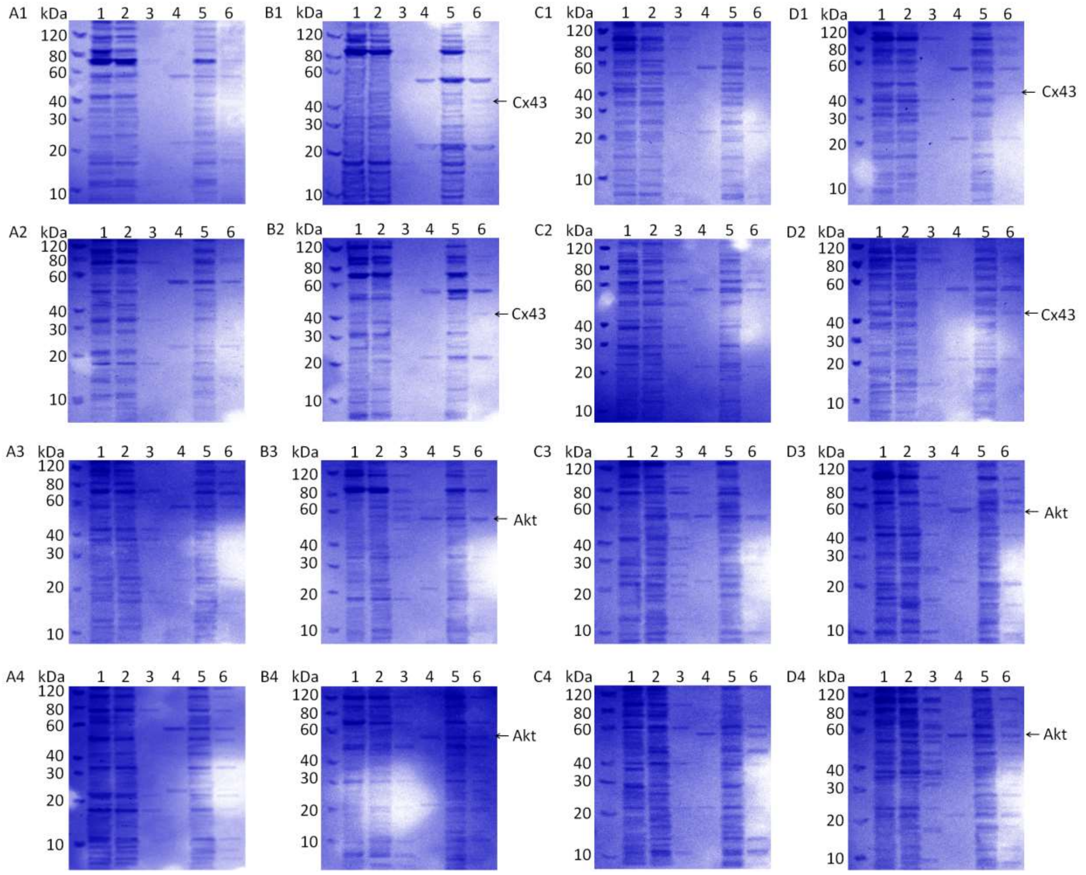

Figure S5. SDS-PAGE of immunoprecipitation analysis in parental cells. Arrow indicates the band of Cx43. Lane 1: total protein of cell lysates; Lane 2: supernatants of total protein incubated with rabbit IgG and protein A/G PLUS-Agarose; Lane 3: precipitations of total protein incubated with rabbit IgG and protein A/G PLUS-Agarose; Lane 4: anti-Akt antibody is used for A1, A2, B1, B2, C1, C2, D1, D2, and anti-Cx43 antibody is used for A3, A4, B3, B4, C3, C4, D3, D4; Lane 5: supernatants of lane 2 + antibody used in lane 4 + protein A/G PLUS-Agarose; Lane 6:

resuspended immunoprecipitates. Molecular weight of Akt and IgG heavy chain is similar, so they are hardly to be discriminated. (A1, 3) Non-treated HCT116 cells. (A2, 4) Non-treated CT26 cells. (B1, 3) Resveratrol-treated HCT116 cells. (B2, 4) Resveratrol-treated CT26 cells. (C1, 3) Cetuximab-treated HCT116 cells. (C2, 4) Cetuximab-treated CT26 cells. (D1, 3) Cetuximab + Resveratrol-treated HCT116 cells. (D2, 4) Cetuximab + Resveratrol-treated CT26 cells.

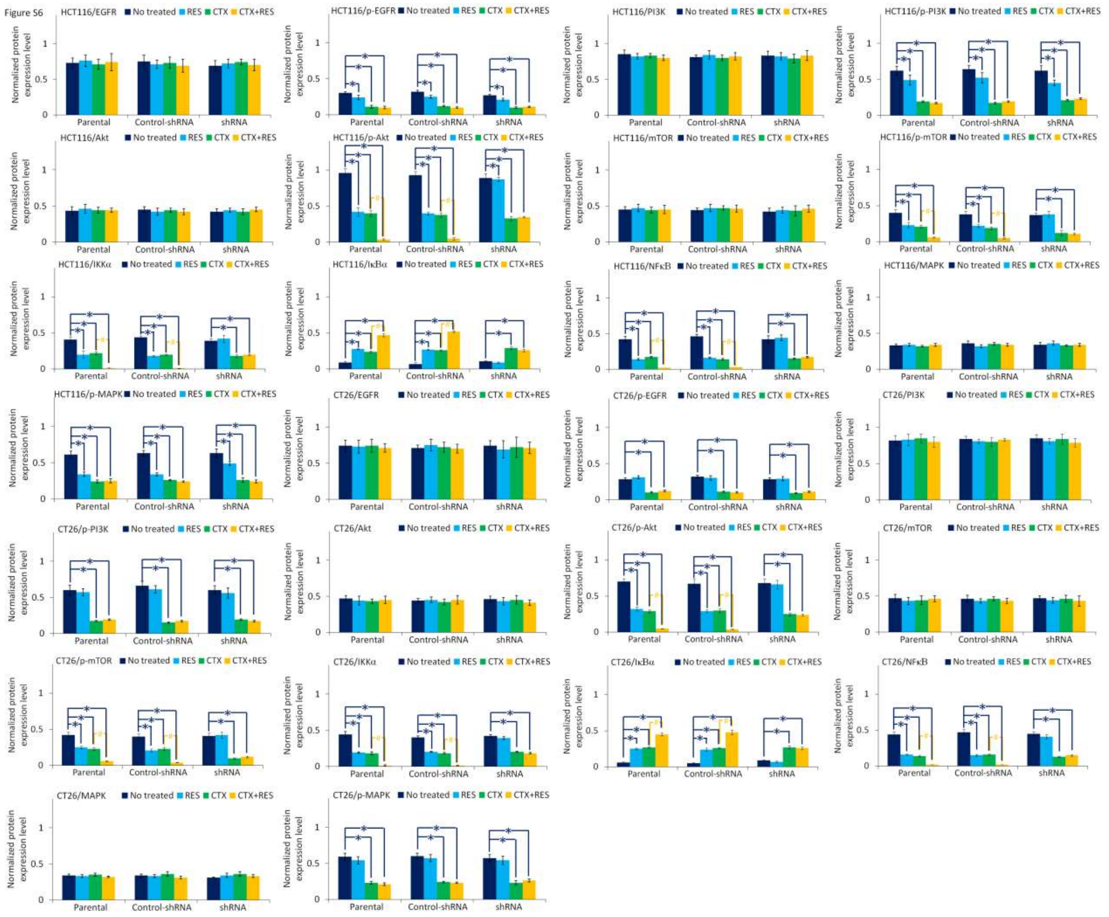

Figure S6. Bar diagrams of densitometric analysis of western blot analysis in Figure 4. “RES” represents resveratrol treatment, “CTX” represents cetuximab treatment, “CTX+RES” represent combination treatment. Groups “CTX”, “RES”, and “CTX+RES” were compared with group “No treated” using one-way ANOVA. Blue **P*<0.05 represents a significant difference. Group “CTX+RES” was compared with

group “CTX” using one-way ANOVA. Yellow *P<0.05 represents a significant difference.

Figure S7A

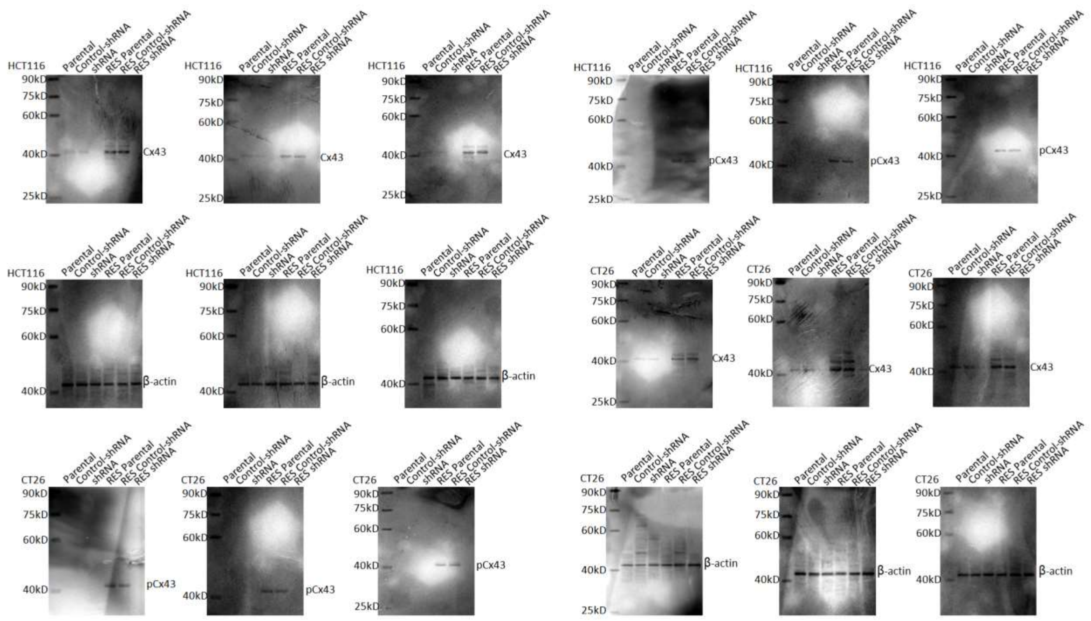

Figure S7B

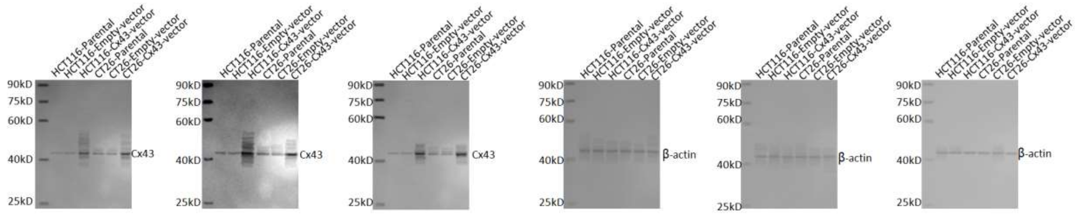

Figure S7C

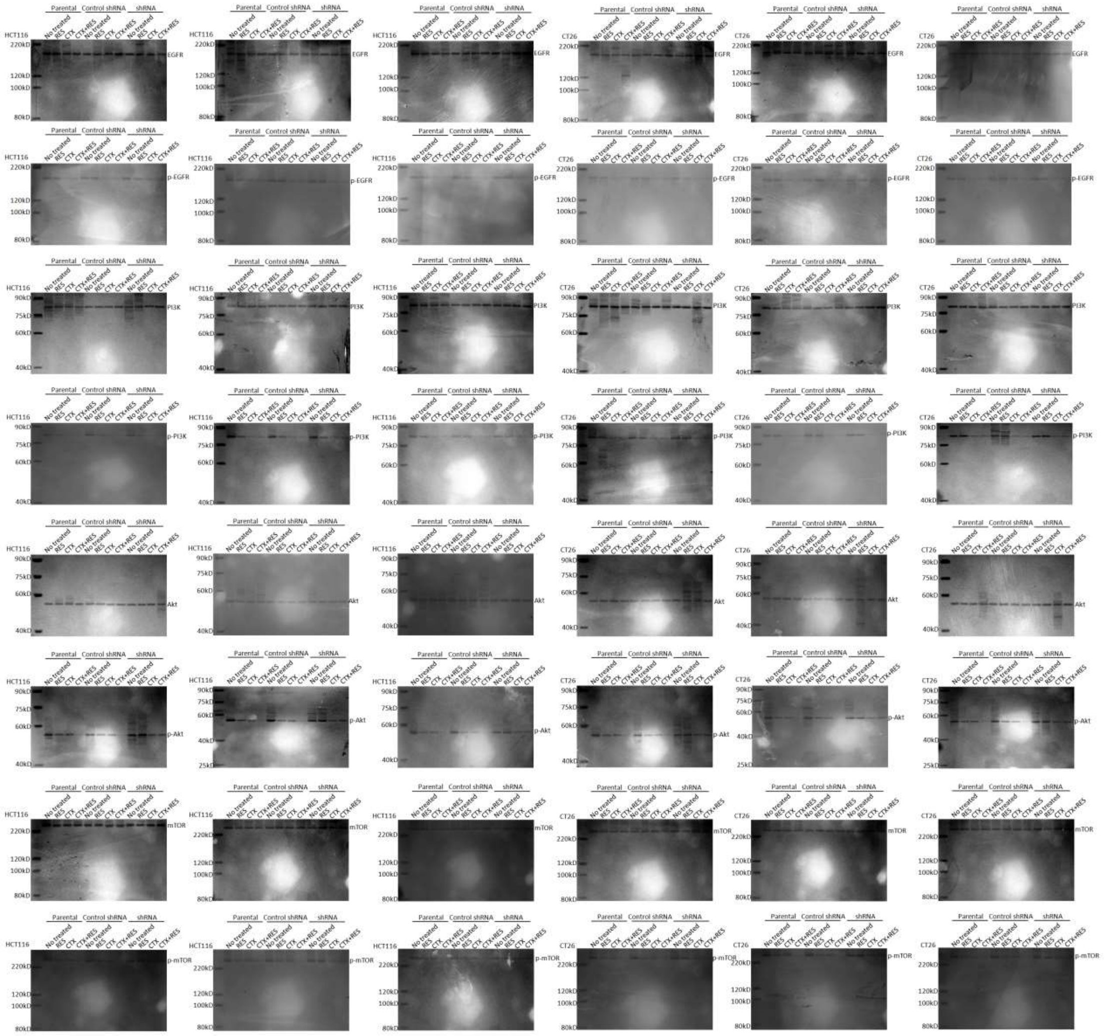

Parental
Control shRNA
shRNA
No treated
RES
CTX
CTX+RES
HCT116
CT26
220kD
120kD
100kD
90kD
80kD
75kD
60kD
40kD
25kD
EGFR
p-EGFR
PI3K
p-PI3K
Akt
p-Akt
mTOR
p-mTOR

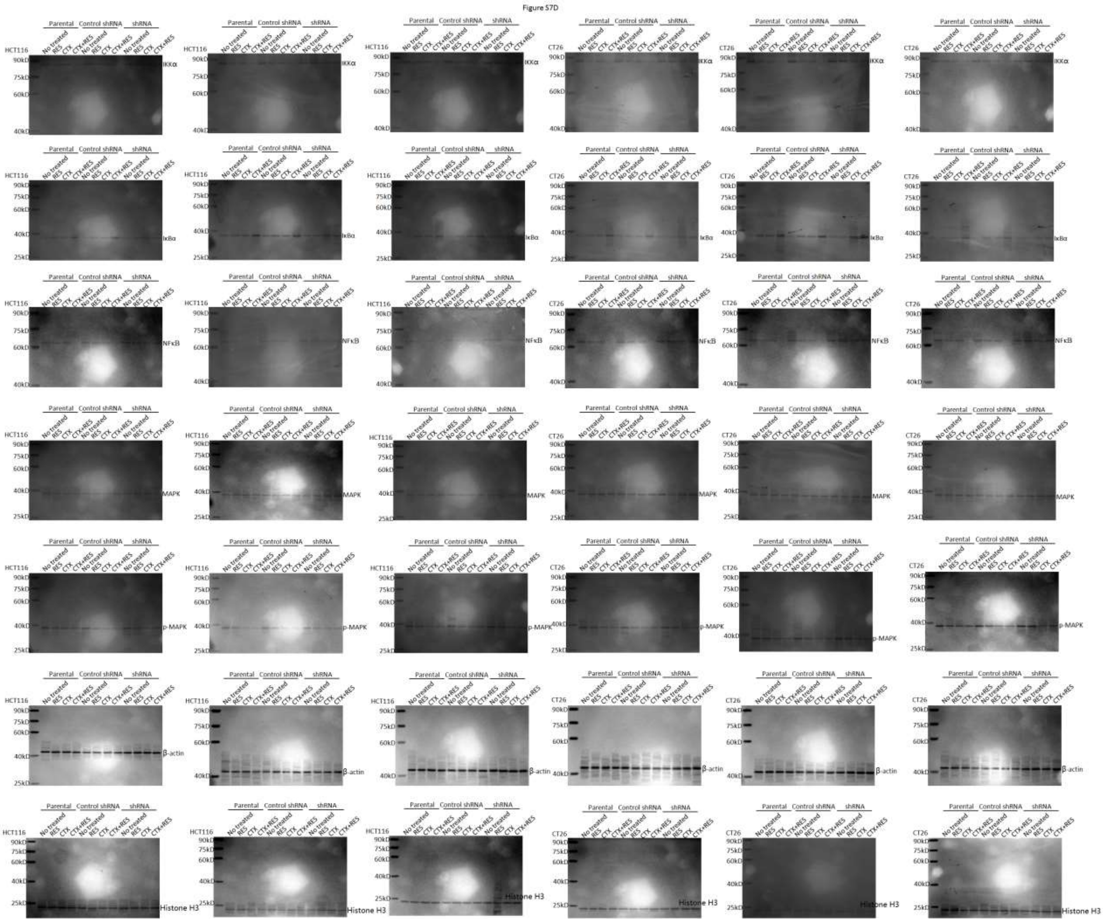

Figure S7. Full length blots of western blot results. “CTX” represents cetuximab treatment, “RES” represents resveratrol treatment.

Table S1. The raw data used for Calcusyn analysis

CTX(μg/mL)	OD490	OD490	OD490	RES(μg/mL)	OD490	OD490	OD490	CTX+RES	OD490	OD490	OD490
**High HCT116**											
0	799	812	786	0	781	796	803		788	806	805
0.2	789	805	779	0.1	751	771	779		758	778	769
1	787	807	781	0.5	702	728	739		707	730	695
2	770	788	782	1	648	679	699		649	651	623
4	751	781	774	2	606	659	680		539	577	477
10	702	768	727	5	556	606	626		458	468	417
20	649	729	683	10	448	495	502		353	397	309
40	475	547	515	20	246	326	363		231	265	166
100	269	330	352	50	85	133	164		139	123	70
**High HCT116-control-shRNA**											
0	800	793	790	0	787	804	778		789	785	803
0.2	790	787	783	0.1	764	779	759		760	757	774
1	794	785	782	0.5	715	742	718		690	701	702
2	773	770	773	1	640	690	644		634	609	644
4	760	769	765	2	611	622	612		502	509	565
10	715	732	733	5	554	570	547		414	404	423
20	672	637	630	10	447	474	447		336	328	317
40	481	446	451	20	291	294	293		198	235	240
100	340	383	353	50	186	168	179		100	122	113
**High HCT116-shRNA**											
0	804	784	799	0	789	798	792		800	809	792
0.2	794	780	795	0.1	759	770	767		777	787	761
1	793	778	793	0.5	703	736	724		709	731	734
2	773	762	778	1	655	689	680		661	667	688
4	766	759	767	2	571	677	662		601	597	611
10	724	732	737	5	539	623	613		538	546	426
20	654	672	702	10	434	501	476		387	385	313
40	473	497	509	20	258	356	319		298	312	243
100	262	327	315	50	79	154	163		173	189	96
**High CT26**											
0	765	758	753	0	768	766	755		754	768	756
0.2	755	752	749	0.1	735	737	726		717	731	712
1	751	751	746	0.5	669	676	665		642	659	607
2	722	726	721	1	592	631	625		579	589	551
4	677	706	710	2	518	558	556		511	508	426
10	658	662	664	5	372	405	403		340	356	281
20	597	624	634	10	258	295	301		265	279	142
40	437	464	464	20	170	297	303		76	224	97
100	205	287	262	50	6	140	163		25	182	32
**High CT26-control-shRNA**											
0	768	753	762	0	756	766	796		751	758	758
0.2	761	748	758	0.1	721	736	763		719	715	698
1	755	746	753	0.5	648	693	709		630	626	638
2	730	717	730	1	585	610	663		547	563	538
4	698	695	703	2	527	539	573		471	478	467
10	676	654	666	5	308	447	476		315	336	302
20	613	621	635	10	250	342	296		253	218	171
40	446	478	440	20	193	285	282		103	125	135
100	195	277	303	50	56	94	139		96	56	45

Continued

Table S1. Continued

CTX(μg/mL)	OD490	OD490	OD490	RES(μg/mL)	OD490	OD490	OD490	CTX+RES	OD490	OD490	OD490
**High CT26-shRNA**											
0	758	774	760	0	760	753	744		756	759	755
0.2	752	770	755	0.1	736	731	723		737	740	731
1	750	767	753	0.5	703	692	687		698	685	667
2	728	742	725	1	631	651	644		656	638	619
4	705	713	701	2	620	576	577		586	566	530
10	680	701	649	5	604	515	544		464	488	444
20	613	653	642	10	485	424	420		406	408	274
40	478	494	446	20	332	266	270		276	283	219
100	306	239	247	50	208	67	43		167	155	78
**Low HCT116**											
0	135	128	133	0	146	135	140		134	130	134
0.2	133	127	132	0.1	142	131	134		128	124	130
1	133	126	132	0.5	134	122	126		119	111	114
2	131	125	131	1	122	115	118		105	101	111
4	130	122	129	2	118	112	112		89	90	92
10	123	116	123	5	113	96	108		77	75	74
20	117	109	112	10	83	81	79		54	62	54
40	90	79	82	20	50	47	50		40	33	31
100	56	48	44	50	23	14	20		21	22	14
**Low HCT116-control-shRNA**											
0	131	125	136	0	126	128	128		122	119	119
0.2	129	124	135	0.1	121	124	124		118	115	114
1	129	123	134	0.5	113	117	117		109	106	99
2	127	121	131	1	101	108	109		99	97	87
4	126	119	130	2	93	102	102		83	82	74
10	119	112	125	5	83	92	91		67	65	56
20	107	102	110	10	65	75	75		54	50	45
40	83	67	74	20	39	50	53		36	35	30
100	54	59	65	50	18	33	34		19	22	8
**Low HCT116-shRNA**											
0	133	130	122	0	129	137	141		136	135	131
0.2	131	129	121	0.1	125	130	136		130	130	128
1	131	129	120	0.5	118	121	129		122	121	120
2	127	127	117	1	113	115	123		112	111	114
4	127	126	116	2	107	104	116		98	99	104
10	120	121	111	5	100	96	109		82	82	87
20	110	114	99	10	81	74	87		57	56	65
40	80	86	73	20	57	47	57		47	45	51
100	47	58	43	50	26	15	27		22	22	34
**Low CT26**											
0	125	127	134	0	119	120	125		134	121	133
0.2	124	125	132	0.1	115	114	119		126	114	127
1	124	125	132	0.5	107	103	108		110	100	113
2	120	120	127	1	101	92	98		98	89	106
4	117	115	121	2	89	83	87		80	73	91
10	111	109	115	5	66	60	63		53	49	61
20	105	102	106	10	50	42	44		37	32	46
40	79	71	77	20	47	36	38		15	18	35
100	53	31	41	50	24	13	15		11	9	23

Continued

Table S1. Continued

CTX(μg/mL)	OD490	OD490	OD490	RES(μg/mL)	OD490	OD490	OD490	CTX+RES	OD490	OD490	OD490
**Low CT26-control-shRNA**											
0	115	120	118	0	119	114	125		121	117	128
0.2	113	119	117	0.1	111	109	120		115	107	122
1	112	119	117	0.5	101	102	113		103	90	108
2	109	114	113	1	90	94	105		89	76	96
4	103	111	109	2	76	83	91		80	62	82
10	96	107	103	5	59	66	69		54	42	59
20	91	102	100	10	38	46	50		35	27	37
40	61	74	75	20	29	38	41		22	14	28
100	33	46	45	50	8	18	19		16	3	17
**Low CT26-control-shRNA**											
0	124	109	128	0	115	120	125		117	119	118
0.2	122	108	127	0.1	110	117	122		113	116	113
1	122	108	127	0.5	104	112	117		104	111	105
2	117	104	122	1	95	103	110		97	104	97
4	112	101	119	2	83	96	100		83	94	85
10	108	97	116	5	76	89	94		69	80	71
20	99	93	111	10	59	74	75		53	62	54
40	68	69	85	20	37	49	52		38	47	38
100	33	38	48	50	10	19	21		16	31	15
**HCT116-empty-vector**											
0	781	793	776	0	802	785	784		781	782	775
0.2	771	789	770	0.1	751	763	762		754	757	747
1	773	781	772	0.5	709	720	734		709	699	676
2	758	779	771	1	670	679	689		663	627	613
4	739	756	762	2	612	654	663		518	576	468
10	689	754	720	5	567	570	628		410	467	406
20	627	697	664	10	441	474	502		356	370	285
40	470	528	498	20	238	341	327		247	271	151
100	274	337	339	50	79	151	170		136	108	79
**HCT116-Cx43-vector**											
0	779	792	785	0	784	784	788		790	771	798
0.2	709	749	751	0.1	743	765	762		673	721	739
1	666	682	693	0.5	685	725	743		587	626	644
2	602	630	577	1	636	693	697		422	494	522
4	437	479	522	2	595	632	660		349	318	389
10	331	319	338	5	523	544	637		218	198	234
20	218	210	233	10	410	453	491		146	82	117
40	120	120	115	20	201	329	320		82	64	48
100	49	45	57	50	98	170	193		52	10	21
**CT26-empty-vector**											
0	784	772	789	0	792	785	796		801	781	776
0.2	773	768	783	0.1	755	752	770		765	746	728
1	768	766	785	0.5	705	688	710		692	659	640
2	751	738	752	1	605	660	647		625	605	577
4	684	718	748	2	511	579	602		523	545	421
10	669	666	688	5	346	423	430		358	357	262
20	608	654	660	10	252	333	360		292	306	150
40	430	466	463	20	163	284	297		91	238	104
100	191	275	317	50	27	149	183		50	176	40

Continued

Table S1. Continued

CTX(μg/mL)	OD490	OD490	OD490	RES(μg/mL)	OD490	OD490	OD490	CTX+RES	OD490	OD490	OD490
**CT26-Cx43-vector**											
0	778	790	789	0	792	803	780		790	792	799
0.2	655	689	661	0.1	751	769	753		595	618	640
1	566	627	597	0.5	700	707	703		432	540	539
2	403	528	537	1	615	685	643		265	399	402
4	269	363	326	2	517	599	603		188	273	329
10	143	309	224	5	355	443	437		106	187	187
20	88	155	163	10	243	344	368		66	121	105
40	59	82	116	20	171	305	308		44	77	59
100	27	26	54	50	47	163	206		37	26	18
**Inhibitor HCT116**											
0	802	793	778	0	785	781	790		792	807	776
0.2	797	787	770	0.1	764	758	769		756	772	741
1	795	784	771	0.5	726	717	716		710	689	670
2	794	772	746	1	678	675	668		632	684	601
4	788	761	729	2	654	631	581		557	535	460
10	745	747	677	5	625	571	543		472	480	402
20	710	704	648	10	474	504	470		407	328	297
40	507	524	447	20	339	303	242		265	280	160
100	345	299	275	50	144	121	98		139	129	68
**Inhibitor HCT116-control-shRNA**											
0	772	796	803	0	801	809	790		793	785	798
0.2	767	788	797	0.1	776	780	764		767	755	770
1	768	789	793	0.5	724	758	722		716	693	695
2	755	776	774	1	684	715	652		629	647	628
4	744	770	758	2	639	612	594		518	536	529
10	717	741	704	5	583	585	580		438	434	405
20	589	631	655	10	475	481	442		348	295	327
40	428	433	509	20	269	306	274		273	203	218
100	338	379	327	50	174	173	199		93	144	85
**Inhibitor HCT116-shRNA**											
0	791	797	805	0	788	802	780		805	778	788
0.2	787	789	797	0.1	767	770	755		781	753	763
1	787	792	793	0.5	724	745	690		734	677	728
2	769	769	776	1	670	702	666		677	625	692
4	754	768	765	2	667	688	546		606	583	625
10	728	738	722	5	592	636	529		533	535	406
20	678	676	649	10	491	483	439		357	383	319
40	526	484	462	20	345	377	261		302	304	257
100	326	318	269	50	144	166	99		175	161	116
**Inhibitor CT26**											
0	770	752	760	0	752	763	759		755	756	747
0.2	764	748	751	0.1	726	734	723		722	717	707
1	765	744	747	0.5	660	670	670		640	650	618
2	732	722	720	1	614	637	591		570	585	549
4	729	701	674	2	548	538	522		516	526	417
10	682	662	668	5	398	424	345		357	367	297
20	644	633	608	10	306	265	257		298	283	147
40	468	457	428	20	268	258	157		231	87	100
100	256	295	214	50	181	155	72		185	21	28

Continued

Table S1. Continued

CTX(μg/mL)	OD490	OD490	OD490	RES(μg/mL)	OD490	OD490	OD490	CTX+RES	OD490	OD490	OD490
**Inhibitor CT26-control-shRNA**											
0	752	746	752	0	743	750	766		742	739	751
0.2	747	742	745	0.1	709	723	733		707	684	707
1	744	737	741	0.5	659	684	662		629	631	603
2	722	713	717	1	610	625	589		548	547	568
4	696	685	675	2	525	531	523		478	461	470
10	654	657	657	5	426	422	301		335	308	351
20	622	624	579	10	268	326	273		258	186	218
40	447	464	430	20	236	253	210		121	128	105
100	286	259	207	50	146	82	64		83	34	74
**Inhibitor CT26-shRNA**											
0	752	755	742	0	757	761	739		742	744	750
0.2	746	751	738	0.1	735	735	713		721	727	717
1	747	749	735	0.5	701	700	682		667	702	647
2	719	718	711	1	659	649	625		635	655	596
4	691	695	693	2	593	591	612		552	583	517
10	637	677	655	5	564	517	579		473	435	424
20	633	647	598	10	439	440	458		393	383	285
40	453	483	461	20	266	274	315		278	270	232
100	239	224	286	50	55	85	215		135	150	108

The concentration of "CTX+RES" is the same to the concentration of CTX and RES in the same line.

Table S2. The statistical difference of growth inhibition between parental and Cx43-shRNA transfected cells in different concentration calculated by one-way ANOVA, *P < 0.05.

	CTX 0.2	CTX 1	CTX 2	CTX 4	CTX 10	CTX 20	CTX 40	CTX 100
High HCT116			*					
High CT26								
Low HCT116		*						
Low CT26								
Inhibitor HCT116			*					
Inhibitor CT26	*	*						

	RES 0.1	RES 0.5	RES 1	RES 2	RES 5	RES 10	RES 20	RES 50
High HCT116								
High CT26	*	*	*	*	*	*		
Low HCT116							*	
Low CT26	*	*			*	*		
Inhibitor HCT116								
Inhibitor CT26		*	*	*	*	*	*	

	CTX 0.2+RES 0.1	CTX 1+RES 0.5	CTX 2+RES 1	CTX 4+RES 2	CTX 10+RES 5	CTX 20+RES 10	CTX 40+RES 20	CTX 100+RES 50
High HCT116	*		*	*	*		*	*
High CT26	*	*	*	*	*	*	*	
Low HCT116	*	*	*	*	*		*	
Low CT26	*	*	*	*	*	*		
Inhibitor HCT116	*	*	*	*	*		*	*
Inhibitor CT26	*	*	*	*	*	*	*	

CTX represents cetuximab, RES represents resveratrol, the numbers represents μg/mL concentration.

Doc.1. The data generated by Calcusyn analysis.

Title:

Date:

Investigator:

Filename: High-HCT116-CTX+RES.csn

Summary table

Drug Combination Index Values at

Drug	ED50	ED75	ED90	Dm	m	r
cetuximab	N/A	N/A	N/A	112.22347	0.87902	0.96080
(Not a combination)						
resveratrol	N/A	N/A	N/A	11.62714	0.76427	0.97993
(Not a combination)						
combination	0.62042	0.60360	0.59054	11.95107	0.79890	0.99719
(2:1)						

Drug: cetuximab

Data points entered:

cetuximab µg/mL	Fraction affected
0.2	0.0093
1	0.0083
2	0.0233
4	0.0370
10	0.0830
20	0.1397
40	0.3583

100 0.6023

Median-effect parameters:

cetuximab

μg/mL

Dm: 112.22347

lower 95%: 49.69084

upper 95%: 253.44925

m: 0.87902 +/- 0.103555

y-int: -1.8021 +/- 0.116935

r: 0.96080

CCSI: -0.67605

Drug: resveratrol

Data points entered:

resveratrol μg/mL	Fraction affected
0.1	0.0327
0.5	0.0833
1	0.1483
2	0.1823
5	0.2483
10	0.3923
20	0.6073
50	0.8393

Median-effect parameters:

resveratrol

μg/mL

Dm: 11.62714

lower 95%: 7.91012

upper 95%: 17.09083

m: 0.76427 +/- 0.063477

y-int: -0.81431 +/- 0.060424

r: 0.97993

CCSI: -0.48573

Drug: combination

Combination: cetuximab resveratrol

Ratio: 2:1

Data points entered:

cetuximab (μg/mL)	resveratrol (μg/mL)	Fraction affected
0.2	0.1	0.0383
1	0.5	0.1107
2	1	0.1977
4	2	0.3350
10	5	0.4393
20	10	0.5583
40	20	0.7233
100	50	0.8607

Median-effect parameters:

cetuximab resveratrol

(µg/mL) (µg/mL)

Dm: 11.95107 5.97553

lower 95%: 10.56687 5.28344

upper 95%: 13.51659 6.75829

m: 0.79890 +/- 0.024502

y-int: -0.86074 +/- 0.027668

r: 0.99719

CCSI: -0.67605

Title:

Date:

Investigator:

Filename: High-HCT116-control-shRNA-CTX+RES.csn

Summary table

Drug Combination Index Values at

Drug	ED50	ED75	ED90	Dm	m	r
cetuximab	N/A	N/A	N/A	101.25327	0.88763	0.96474
(Not a combination)						
resveratrol	N/A	N/A	N/A	11.49965	0.75105	0.99428
(Not a combination)						
combination	0.60799	0.57787	0.55405	11.39502	0.80409	0.99856
(2:1)						

Drug: cetuximab

Data points entered:

cetuximab Fraction affected

µg/mL

0.2	0.0090
1	0.0083
2	0.0273
4	0.0367
10	0.0847
20	0.1860
40	0.4210
100	0.5480

Median-effect parameters:

cetuximab

μg/mL

Dm: 101.25327

lower 95%: 47.75686

upper 95%: 214.67544

m: 0.88763 +/- 0.098869

y-int: -1.7801 +/- 0.111644

r: 0.96474

CCSI: -0.67605

Drug: resveratrol

Data points entered:

resveratrol μg/mL	Fraction affected
0.1	0.0280
0.5	0.0813
1	0.1660

2	0.2207
5	0.2943
10	0.4220
20	0.6287
50	0.7743

Median-effect parameters:

	resveratrol
	μg/mL
Dm:	11.49965
lower 95%:	9.38807
upper 95%:	14.08618

m: 0.75105 +/- 0.032944

y-int: -0.79663 +/- 0.031359

r: 0.99428

CCSI: -0.48573

Drug: combination

Combination: cetuximab resveratrol

Ratio: 2:1

Data points entered:

cetuximab (μg/mL)	resveratrol (μg/mL)	Fraction affected
0.2	0.1	0.0357
1	0.5	0.1187
2	1	0.2057
4	2	0.3367

10	5	0.4777
20	10	0.5860
40	20	0.7163
100	50	0.8583

Median-effect parameters:

	cetuximab (μg/mL)	resveratrol (μg/mL)
Dm:	11.39502	5.69751
lower 95%:	10.44239	5.22119
upper 95%:	12.43456	6.21728

m: 0.80409 +/- 0.017631

y-int: -0.84969 +/- 0.019909

r: 0.99856

CCSI: -0.67605

Title:

Date:

Investigator:

Filename: High-HCT116-shRNA-CTX+RES.csn

Summary table

Drug Combination Index Values at

Drug	ED50	ED75	ED90	Dm	m	r
cetuximab	N/A	N/A	N/A	96.02317	0.91250	0.97311
(Not a combination)						
resveratrol	N/A	N/A	N/A	11.39517	0.75156	0.98246
(Not a combination)						
combination	0.93164	0.92656	0.93258	17.15968	0.78393	0.99922

(2:1)

Drug: cetuximab

Data points entered:

cetuximab µg/mL	Fraction affected
0.2	0.0070
1	0.0090
2	0.0303
4	0.0393
10	0.0807
20	0.1497
40	0.3797
100	0.6200

Median-effect parameters:

cetuximab

µg/mL

Dm: 96.02317

lower 95%: 50.44833

upper 95%: 182.77018

m: 0.91250 +/- 0.088177

y-int: -1.8089 +/- 0.099570

r: 0.97311

CCSI: -0.67605

Drug: resveratrol

Data points entered:

resveratrol µg/mL	Fraction affected
0.1	0.0340
0.5	0.0900
1	0.1487
2	0.1970
5	0.2536
10	0.4063
20	0.6073
50	0.8327

Median-effect parameters:

	resveratrol µg/mL
Dm:	11.39517
lower 95%:	7.96922
upper 95%:	16.29392

m: 0.75156 +/- 0.058239

y-int: -0.79419 +/- 0.055438

r: 0.98246

CCSI: -0.48573

Drug: combination

Combination: cetuximab resveratrol

Ratio: 2:1

Data points entered:

cetuximab (μg/mL)	resveratrol (μg/mL)	Fraction affected
0.2	0.1	0.0306
1	0.5	0.0937
2	1	0.1597
4	2	0.2457
10	5	0.3713
20	10	0.5477
40	20	0.6447
100	50	0.8090

Median-effect parameters:

	cetuximab (μg/mL)	resveratrol (μg/mL)
Dm:	17.15968	8.57984
lower 95%:	16.00915	8.00458
upper 95%:	18.39290	9.19645

m: 0.78393 +/- 0.012608

y-int: -0.96776 +/- 0.014237

r: 0.99922

CCSI: -0.67605

Title:

Date:

Investigator:

Filename: High-CT26-CTX+RES.csn

Summary table

Drug Combination Index Values at

Drug	ED50	ED75	ED90	Dm	m	r
cetuximab (Not a combination)	N/A	N/A	N/A	69.41670	0.89583	0.98066
resveratrol (Not a combination)	N/A	N/A	N/A	5.94773	0.79365	0.99656
combination (2:1)	0.70713	0.67955	0.65532	7.18111	0.83244	0.99925

Drug: cetuximab

Data points entered:

cetuximab µg/mL	Fraction affected
0.2	0.0083
1	0.0120
2	0.0460
4	0.0800
10	0.1273
20	0.1843
40	0.3993
100	0.6680

Median-effect parameters:

	cetuximab µg/mL
Dm:	69.41670
lower 95%:	42.07219
upper 95%:	114.53357

m: 0.89583 +/- 0.072988

y-int: -1.6496 +/- 0.082418

r: 0.98066

CCSI: -0.67605

Drug: resveratrol

Data points entered:

resveratrol µg/mL	Fraction affected
0.1	0.0390
0.5	0.1213
1	0.1920
2	0.2863
5	0.4840
10	0.6263
20	0.6627
50	0.8637

Median-effect parameters:

	resveratrol µg/mL
Dm:	5.94773
lower 95%:	5.19108
upper 95%:	6.81468

m: 0.79365 +/- 0.026927

y-int: -0.61456 +/- 0.025632

r: 0.99656

CCSI: -0.48573

Drug: combination

Combination: cetuximab resveratrol

Ratio: 2:1

Data points entered:

cetuximab (μg/mL)	resveratrol (μg/mL)	Fraction affected
0.2	0.1	0.0510
1	0.5	0.1617
2	1	0.2450
4	2	0.3650
10	5	0.5710
20	10	0.6983
40	20	0.8260
100	50	0.8950

Median-effect parameters:

	cetuximab (μg/mL)	resveratrol (μg/mL)
Dm:	7.18111	3.59055
lower 95%:	6.76415	3.38207
upper 95%:	7.62378	3.81189

m: 0.83244 +/- 0.013179

y-int: -0.71273 +/- 0.014881

r: 0.99925

CCSI: -0.67605

Title:

Date:

Investigator:

Filename: High-CT26-control-shRNA-CTX+RES.csn

Summary table

Drug Combination Index Values at

Drug	ED50	ED75	ED90	Dm	m	r
cetuximab (Not a combination)	N/A	N/A	N/A	68.24735	0.91766	0.98427
resveratrol (Not a combination)	N/A	N/A	N/A	5.81648	0.79829	0.99617
combination (2:1)	0.64388	0.61987	0.59945	6.39946	0.83860	0.99866

Drug: cetuximab

Data points entered:

cetuximab µg/mL	Fraction affected
0.2	0.0067
1	0.0117
2	0.0457
4	0.0810
10	0.1250
20	0.1807
40	0.4017
100	0.6593

Median-effect parameters:

cetuximab

μg/mL

Dm: 68.24735

lower 95%: 43.58582

upper 95%: 106.86278

m: 0.91766 +/- 0.067240

y-int: -1.6831 +/- 0.075928

r: 0.98427

CCSI: -0.67605

Drug: resveratrol

Data points entered:

resveratrol μg/mL	Fraction affected
0.1	0.0413
0.5	0.1153
1	0.1980
2	0.2927
5	0.4697
10	0.6163
20	0.6720
50	0.8753

Median-effect parameters:

resveratrol

μg/mL

Dm: 5.81648

lower 95%: 5.04022

upper 95%: 6.71230

m: 0.79829 +/- 0.028621

y-int: -0.61042 +/- 0.027245

r: 0.99617

CCSI: -0.48573

Drug: combination

Combination: cetuximab resveratrol

Ratio: 2:1

Data points entered:

cetuximab (μg/mL)	resveratrol (μg/mL)	Fraction affected
0.2	0.1	0.0587
1	0.5	0.1640
2	1	0.2720
4	2	0.3747
10	5	0.5790
20	10	0.7163
40	20	0.8393
100	50	0.9123

Median-effect parameters:

	cetuximab	resveratrol
	(μg/mL)	(μg/mL)

Dm: 6.39946 3.19973

lower 95%: 5.91073 2.95536

upper 95%: 6.92860 3.46430

m: 0.83860 +/- 0.017718

y-int: -0.67603 +/- 0.020007

r: 0.99866

CCSI: -0.67605

Title:

Date:

Investigator:

Filename: High-CT26-shRNA-CTX+RES.csn

Summary table

Drug Combination Index Values at

	ED50	ED75	ED90	Dm	m	r
cetuximab	N/A	N/A	N/A	72.91511	0.93855	0.97948
(Not a combination)						
resveratrol	N/A	N/A	N/A	10.33991	0.80367	0.98275
(Not a combination)						
combination	1.02721	1.06991	1.12261	16.54902	0.80728	0.99917
(2:1)						

Drug: cetuximab

Data points entered:

cetuximab Fraction affected

μg/mL

0.2 0.0060

1	0.0087
2	0.0417
4	0.0747
10	0.1140
20	0.1670
40	0.3807
100	0.6533

Median-effect parameters:

	cetuximab
	µg/mL
Dm:	72.91511
lower 95%:	43.22985
upper 95%:	122.98480

m: 0.93855 +/- 0.078849

y-int: -1.7483 +/- 0.089037

r: 0.97948

CCSI: -0.67605

Drug: resveratrol

Data points entered:

resveratrol µg/mL	Fraction affected
0.1	0.0290
0.5	0.0770
1	0.1460
2	0.2143

5	0.2630
10	0.4107
20	0.6150
50	0.8593

Median-effect parameters:

resveratrol

µg/mL

Dm: 10.33991

lower 95%: 7.31352

upper 95%: 14.61864

m: 0.80367 +/- 0.061752

y-int: -0.81533 +/- 0.058782

r: 0.98275

CCSI: -0.48573

Drug: combination

Combination: cetuximab resveratrol

Ratio: 2:1

Data points entered:

cetuximab (µg/mL)	resveratrol (µg/mL)	Fraction affected
0.2	0.1	0.0267
1	0.5	0.0963
2	1	0.1567
4	2	0.2587
10	5	0.3847

20	10	0.5203
40	20	0.6567
100	50	0.8233

Median-effect parameters:

	cetuximab (μg/mL)	resveratrol (μg/mL)
Dm:	16.54902	8.27451
lower 95%:	15.41344	7.70672
upper 95%:	17.76825	8.88413

m: 0.80728 +/- 0.013406

y-int: -0.9839 +/- 0.015138

r: 0.99917

CCSI: -0.67605

Title:

Date:

Investigator:

Filename: Low-HCT116-CTX+RES.csn

Summary table

Drug	Combination Index Values at ED50	ED75	ED90	Dm	m	r
cetuximab (Not a combination)	N/A	N/A	N/A	97.52552	0.94929	0.97453
resveratrol (Not a combination)	N/A	N/A	N/A	10.16198	0.79468	0.97736
combination (2:1)	0.70514	0.73614	0.77491	11.85979	0.79446	0.99828

Drug: cetuximab

Data points entered:

cetuximab µg/mL	Fraction affected
0.2	0.0060
1	0.0077
2	0.0203
4	0.0353
10	0.0820
20	0.1430
40	0.3627
100	0.6200

Median-effect parameters:

	cetuximab µg/mL
Dm:	97.52552
lower 95%:	52.03750
upper 95%:	182.77641

m: 0.94929 +/- 0.089187

y-int: -1.8882 +/- 0.100710

r: 0.97453

CCSI: -0.67605

Drug: resveratrol

Data points entered:

resveratrol   Fraction affected

µg/mL

0.1	0.0317
0.5	0.0900
1	0.1517
2	0.1823
5	0.2443
10	0.4200
20	0.6463
50	0.8620

Median-effect parameters:

resveratrol

µg/mL

Dm: 10.16198

lower 95%: 6.83443

upper 95%: 15.10967

m: 0.79468 +/- 0.070239

y-int: -0.80022 +/- 0.066861

r: 0.97736

CCSI: -0.48573

Drug: combination

Combination: cetuximab   resveratrol

Ratio: 2:1

Data points entered:

cetuximab (μg/mL)	resveratrol (μg/mL)	Fraction affected
0.2	0.1	0.0367
1	0.5	0.1287
2	1	0.2013
4	2	0.3150
10	5	0.4297
20	10	0.5677
40	20	0.7370
100	50	0.8543

Median-effect parameters:

	cetuximab (μg/mL)	resveratrol (μg/mL)
Dm:	11.85979	5.92990
lower 95%:	10.77215	5.38608
upper 95%:	13.05725	6.52863

m: 0.79446 +/- 0.019065

y-int: -0.85331 +/- 0.021528

r: 0.99828

CCSI: -0.67605

Title:

Date:

Investigator:

Filename: Low-HCT116-control-shRNA-CTX+RES.csn

Summary table

Drug Combination Index Values at

	ED50	ED75	ED90	Dm	m	r
cetuximab (Not a combination)	N/A	N/A	N/A	98.12426	0.90207	0.97185
resveratrol (Not a combination)	N/A	N/A	N/A	11.28361	0.75138	0.99569
combination (2:1)	0.62477	0.59677	0.57603	11.46301	0.80435	0.99860

Drug: cetuximab

Data points entered:

cetuximab µg/mL	Fraction affected
0.2	0.0077
1	0.0086
2	0.0270
4	0.0377
10	0.0867
20	0.1837
40	0.4247
100	0.5420

Median-effect parameters:

	cetuximab µg/mL
Dm:	98.12426
lower 95%:	50.57940
upper 95%:	190.36150

m: 0.90207 +/- 0.089278

y-int: -1.7967 +/- 0.100814

r: 0.97185

CCSI: -0.67605

Drug: resveratrol

Data points entered:

resveratrol µg/mL	Fraction affected
0.1	0.0277
0.5	0.0870
1	0.1640
2	0.2177
5	0.3010
10	0.4340
20	0.6270
50	0.7753

Median-effect parameters:

	resveratrol µg/mL
Dm:	11.28361
lower 95%:	9.47212
upper 95%:	13.44155

m: 0.75138 +/- 0.028562

y-int: -0.79079 +/- 0.027189

r: 0.99569

CCSI: -0.48573

Drug: combination

Combination: cetuximab resveratrol

Ratio: 2:1

Data points entered:

cetuximab (μg/mL)	resveratrol (μg/mL)	Fraction affected
0.2	0.1	0.0347
1	0.5	0.1223
2	1	0.2083
4	2	0.3303
10	5	0.4737
20	10	0.5827
40	20	0.7153
100	50	0.8587

Median-effect parameters:

	cetuximab (μg/mL)	resveratrol (μg/mL)
Dm:	11.46301	5.73150
lower 95%:	10.51756	5.25878
upper 95%:	12.49345	6.24672

m: 0.80435 +/- 0.017372

y-int: -0.85204 +/- 0.019617

r: 0.99860

CCSI: -0.67605

Title:

Date:

Investigator:

Filename: Low-HCT116-shRNA-CTX+RES.csn

Summary table

Drug Combination Index Values at

Drug	ED50	ED75	ED90	Dm	m	r
cetuximab (Not a combination)	N/A	N/A	N/A	96.84722	0.90121	0.97639
resveratrol (Not a combination)	N/A	N/A	N/A	11.89915	0.74617	0.98131
combination (2:1)	0.91065	0.90420	0.90837	17.39693	0.77920	0.99917

Drug: cetuximab

Data points entered:

cetuximab µg/mL	Fraction affected
0.2	0.0070
1	0.0103
2	0.0317
4	0.0387
10	0.0820
20	0.1573
40	0.3760
100	0.6117

Median-effect parameters:

cetuximab

μg/mL

Dm: 96.84722

lower 95%: 52.99995

upper 95%: 176.96968

m: 0.90121 +/- 0.081397

y-int: -1.7899 +/- 0.091914

r: 0.97639

CCSI: -0.67605

Drug: resveratrol

Data points entered:

resveratrol μg/mL	Fraction affected
0.1	0.0353
0.5	0.0907
1	0.1340
2	0.1923
5	0.2486
10	0.4020
20	0.5997
50	0.8300

Median-effect parameters:

resveratrol

μg/mL

Dm: 11.89915

lower 95%: 8.19106

upper 95%: 17.28589

m: 0.74617 +/- 0.059734

y-int: -0.80251 +/- 0.056861

r: 0.98131

CCSI: -0.48573

Drug: combination

Combination: cetuximab resveratrol

Ratio: 2:1

Data points entered:

cetuximab (μg/mL)	resveratrol (μg/mL)	Fraction affected
0.2	0.1	0.0307
1	0.5	0.0923
2	1	0.1600
4	2	0.2463
10	5	0.3730
20	10	0.5540
40	20	0.6423
100	50	0.7997

Median-effect parameters:

	cetuximab (μg/mL)	resveratrol (μg/mL)
Dm:	17.39693	8.69846

lower 95%: 16.18484 8.09242

upper 95%: 18.69979 9.34989

m: 0.77920 +/- 0.013000

y-int: -0.96658 +/- 0.014680

r: 0.99917

CCSI: -0.67605

Title:

Date:

Investigator:

Filename: Low-CT26-CTX+RES.csn

Summary table

Drug Combination Index Values at

Drug	ED50	ED75	ED90	Dm	m	r
cetuximab	N/A	N/A	N/A	68.79303	0.88867	0.97853
(Not a combination)						
resveratrol	N/A	N/A	N/A	6.14783	0.78495	0.99735
(Not a combination)						
combination	0.69697	0.67064	0.64779	7.27023	0.82321	0.99911
(2:1)						

Drug: cetuximab

Data points entered:

cetuximab µg/mL	Fraction affected
0.2	0.0090
1	0.0120

2 0.0467

4 0.0833

10 0.1297

20 0.1853

40 0.4063

100 0.6697

Median-effect parameters:

cetuximab

μg/mL

Dm: 68.79303

lower 95%: 40.60065

upper 95%: 116.56169

m: 0.88867 +/- 0.076421

y-int: -1.633 +/- 0.086296

r: 0.97853

CCSI: -0.67605

Drug: resveratrol

Data points entered:

resveratrol Fraction affected

μg/mL

0.1 0.0380

0.5 0.1230

1 0.1933

2 0.2843

5 0.4757

10	0.6233
20	0.6620
50	0.8520

Median-effect parameters:

resveratrol

μg/mL

Dm: 6.14783

lower 95%: 5.45202

upper 95%: 6.93244

m: 0.78495 +/- 0.023374

y-int: -0.61911 +/- 0.022250

r: 0.99735

CCSI: -0.48573

Drug: combination

Combination: cetuximab resveratrol

Ratio: 2:1

Data points entered:

cetuximab (μg/mL)	resveratrol (μg/mL)	Fraction affected
0.2	0.1	0.0503
1	0.5	0.1650
2	1	0.2443
4	2	0.3677
10	5	0.5767
20	10	0.7003

40	20	0.8216
100	50	0.8867

Median-effect parameters:

	cetuximab (μg/mL)	resveratrol (μg/mL)
Dm:	7.27023	3.63512
lower 95%:	6.81250	3.40625
upper 95%:	7.75871	3.87936

m: 0.82321 +/- 0.014157

y-int: -0.70924 +/- 0.015987

r: 0.99911

CCSI: -0.67605

Title:

Date:

Investigator:

Filename: Low-CT26-control-shRNA-CTX+RES.csn

Summary table

Drug	Combination Index Values at ED50	ED75	ED90	Dm	m	r
cetuximab (Not a combination)	N/A	N/A	N/A	69.59423	0.92630	0.98423
resveratrol (Not a combination)	N/A	N/A	N/A	5.77327	0.78525	0.99606
combination (2:1)	0.66192	0.64859	0.63961	6.55533	0.81652	0.99951

Drug: cetuximab

Data points entered:

cetuximab µg/mL	Fraction affected
0.2	0.0060
1	0.0110
2	0.0450
4	0.0803
10	0.1280
20	0.1683
40	0.4007
100	0.6473

Median-effect parameters:

cetuximab

µg/mL

Dm: 69.59423

lower 95%: 44.31742

upper 95%: 109.28788

m: 0.92630 +/- 0.067975

y-int: -1.7068 +/- 0.076758

r: 0.98423

CCSI: -0.67605

Drug: resveratrol

Data points entered:

resveratrol µg/mL	Fraction affected
0.1	0.0477
0.5	0.1133
1	0.1893
2	0.3007
5	0.4527
10	0.6230
20	0.6917
50	0.8703

Median-effect parameters:

	resveratrol µg/mL
Dm:	5.77327
lower 95%:	4.99337
upper 95%:	6.67498

m: 0.78525 +/- 0.028559

y-int: -0.59791 +/- 0.027186

r: 0.99606

CCSI: -0.48573

Drug: combination

Combination: cetuximab resveratrol

Ratio: 2:1

Data points entered:

cetuximab resveratrol Fraction affected

(μg/mL)		(μg/mL)
0.2	0.1	0.0560
1	0.5	0.1723
2	1	0.2837
4	2	0.3847
10	5	0.5743
20	10	0.7257
40	20	0.8227
100	50	0.8987

Median-effect parameters:

	cetuximab (μg/mL)	resveratrol (μg/mL)
Dm:	6.55533	3.27766
lower 95%:	6.24837	3.12418
upper 95%:	6.87737	3.43868

m: 0.81652 +/- 0.010407

y-int: -0.66677 +/- 0.011751

r: 0.99951

CCSI: -0.67605

Title:

Date:

Investigator:

Filename: Low-CT26-shRNA-CTX+RES.csn

Summary table

Drug Combination Index Values at

ED50 ED75 ED90 Dm m r

cetuximab N/A N/A N/A 73.59724 0.94206 0.97525

(Not a combination)

resveratrol N/A N/A N/A 10.17224 0.81295 0.98447

(Not a combination)

combination 1.07422 1.14705 1.23270 17.12160 0.79965 0.99909

(2:1)

Drug: cetuximab

Data points entered:

cetuximab µg/mL	Fraction affected
0.2	0.0060
1	0.0081
2	0.0423
4	0.0757
10	0.1073
20	0.1570
40	0.3800
100	0.6637

Median-effect parameters:

cetuximab

µg/mL

Dm: 73.59724

lower 95%: 41.31652

upper 95%: 131.09897

m: 0.94206 +/- 0.087203

y-int: -1.7587 +/- 0.098470

r: 0.97525

CCSI: -0.67605

Drug: resveratrol

Data points entered:

resveratrol µg/mL	Fraction affected
0.1	0.0290
0.5	0.0697
1	0.1430
2	0.2217
5	0.2777
10	0.4183
20	0.6123
50	0.8600

Median-effect parameters:

	resveratrol µg/mL
Dm:	10.17224
lower 95%:	7.33608
upper 95%:	14.10488

m: 0.81295 +/- 0.059192

y-int: -0.81898 +/- 0.056345

r: 0.98447

CCSI: -0.48573

Drug: combination

Combination: cetuximab resveratrol

Ratio: 2:1

Data points entered:

cetuximab (μg/mL)	resveratrol (μg/mL)	Fraction affected
0.2	0.1	0.0280
1	0.5	0.0923
2	1	0.1530
4	2	0.2570
10	5	0.3717
20	10	0.5183
40	20	0.6513
100	50	0.8187

Median-effect parameters:

	cetuximab (μg/mL)	resveratrol (μg/mL)
Dm:	17.12160	8.56080
lower 95%:	15.87909	7.93954
upper 95%:	18.46134	9.23067

m: 0.79965 +/- 0.013968

y-int: -0.98641 +/- 0.015772

r: 0.99909

CCSI: -0.67605

Title:

Date:

Investigator:

Filename: HCT116-empty-vector-CTX+RES.csn

Summary table

Drug	Combination Index Values at					
	ED50	ED75	ED90	Dm	m	r
cetuximab (Not a combination)	N/A	N/A	N/A	107.11325	0.90209	0.96997
resveratrol (Not a combination)	N/A	N/A	N/A	11.69775	0.74044	0.97937
combination (2:1)	0.63306	0.57806	0.53431	12.15574	0.82006	0.99789

Drug: cetuximab

Data points entered:

cetuximab μg/mL	Fraction affected
0.2	0.0077
1	0.0100
2	0.0173
4	0.0387
10	0.0793
20	0.1533
40	0.3630
100	0.5950

Median-effect parameters:

cetuximab

μg/mL

Dm: 107.11325

lower 95%: 53.18748

upper 95%: 215.71334

m: 0.90209 +/- 0.092344

y-int: -1.8311 +/- 0.104276

r: 0.96997

CCSI: -0.67605

Drug: resveratrol

Data points entered:

resveratrol μg/mL	Fraction affected
0.1	0.0393
0.5	0.0867
1	0.1397
2	0.1853
5	0.2547
10	0.4013
20	0.6163
50	0.8303

Median-effect parameters:

resveratrol

μg/mL

Dm: 11.69775

lower 95%: 7.91048

upper 95%: 17.29824

m: 0.74044 +/- 0.062363

y-int: -0.79086 +/- 0.059364

r: 0.97937

CCSI: -0.48573

Drug: combination

Combination: cetuximab resveratrol

Ratio: 2:1

Data points entered:

cetuximab (μg/mL)	resveratrol (μg/mL)	Fraction affected
0.2	0.1	0.0333
1	0.5	0.1077
2	1	0.1853
4	2	0.3317
10	5	0.4507
20	10	0.5670
40	20	0.7133
100	50	0.8613

Median-effect parameters:

	cetuximab (μg/mL)	resveratrol (μg/mL)
Dm:	12.15574	6.07787
lower 95%:	10.92432	5.46216

upper 95%: 13.52596 6.76298

m: 0.82006 +/- 0.021759

y-int: -0.88958 +/- 0.024570

r: 0.99789

CCSI: -0.67605

Title:

Date:

Investigator:

Filename: HCT116-Cx43-vector-CTX+RES.csn

Summary table

Drug	Combination Index Values at					
	ED50	ED75	ED90	Dm	m	r
cetuximab (Not a combination)	N/A	N/A	N/A	7.96164	1.16118	0.98363
resveratrol (Not a combination)	N/A	N/A	N/A	11.39142	0.74514	0.98941
combination (2:1)	0.52541	0.60192	0.71748	3.09983	0.91978	0.99649

Drug: cetuximab

Data points entered:

cetuximab µg/mL	Fraction affected
0.2	0.0062
1	0.1333
2	0.2317

4 0.3897

10 0.5797

20 0.7183

40 0.8487

100 0.9353

Median-effect parameters:

cetuximab

μg/mL

Dm: 7.96164

lower 95%: 5.98902

upper 95%: 10.58398

m: 1.16118 +/- 0.086858

y-int: -1.0462 +/- 0.098081

r: 0.98363

CCSI: -0.67605

Drug: resveratrol

Data points entered:

resveratrol Fraction affected

μg/mL

0.1 0.0360

0.5 0.0860

1 0.1397

2 0.1987

5 0.2760

10 0.4243

20	0.6387
50	0.8033

Median-effect parameters:

	resveratrol
	μg/mL
Dm:	11.39142
lower 95%:	8.64115
upper 95%:	15.01703

m: 0.74514 +/- 0.044621

y-int: -0.7873 +/- 0.042475

r: 0.98941

CCSI: -0.48573

Drug: combination

Combination: cetuximab resveratrol

Ratio: 2:1

Data points entered:

cetuximab (μg/mL)	resveratrol (μg/mL)	Fraction affected
0.2	0.1	0.0950
1	0.5	0.2117
2	1	0.3897
4	2	0.5520
10	5	0.7240
20	10	0.8537
40	20	0.9170

100	50	0.9640

Median-effect parameters:

	cetuximab (μg/mL)	resveratrol (μg/mL)
Dm:	3.09983	1.54992
lower 95%:	2.70708	1.35354
upper 95%:	3.54957	1.77479

m: 0.91978 +/- 0.031540

y-int: -0.45192 +/- 0.035616

r: 0.99649

CCSI: -0.67605

Title:

Date:

Investigator:

Filename: CT26-empty-vector-CTX+RES.csn

Summary table

Drug Combination Index Values at

	ED50	ED75	ED90	Dm	m	r
cetuximab (Not a combination)	N/A	N/A	N/A	67.64049	0.90353	0.97694
resveratrol (Not a combination)	N/A	N/A	N/A	6.12967	0.78644	0.99841
combination (2:1)	0.72572	0.70041	0.67924	7.53176	0.82512	0.99890

Drug: cetuximab

Data points entered:

cetuximab µg/mL	Fraction affected
0.2	0.0087
1	0.0107
2	0.0437
4	0.0823
10	0.1370
20	0.1797
40	0.4197
100	0.6653

Median-effect parameters:

	cetuximab µg/mL
Dm:	67.64049
lower 95%:	39.23108
upper 95%:	116.62273

m:	0.90353 +/- 0.080609
y-int:	-1.6536 +/- 0.091024
r:	0.97694
CCSI:	-0.67605

Drug: resveratrol

Data points entered:

resveratrol Fraction affected

μg/mL

0.1	0.0397
0.5	0.1130
1	0.1933
2	0.2863
5	0.4937
10	0.6010
20	0.6853
50	0.8477

Median-effect parameters:

resveratrol

μg/mL

Dm: 6.12967

lower 95%: 5.58574

upper 95%: 6.72657

m: 0.78644 +/- 0.018127

y-int: -0.61927 +/- 0.017255

r: 0.99841

CCSI: -0.48573

Drug: combination

Combination: cetuximab resveratrol

Ratio: 2:1

Data points entered:

cetuximab (μg/mL)	resveratrol (μg/mL)	Fraction affected

0.2	0.1	0.0497
1	0.5	0.1553
2	1	0.2333
4	2	0.3683
10	5	0.5853
20	10	0.6826
40	20	0.8153
100	50	0.8863

Median-effect parameters:

	cetuximab (μg/mL)	resveratrol (μg/mL)
Dm:	7.53176	3.76588
lower 95%:	7.00527	3.50263
upper 95%:	8.09782	4.04891

m: 0.82512 +/- 0.015777

y-int: -0.72355 +/- 0.017815

r: 0.99890

CCSI: -0.67605

Title:

Date:

Investigator:

Filename: CT26-Cx43-vector-CTX+RES.csn

Summary table

Drug	Combination Index Values at ED50	ED75	ED90	Dm	m	r
cetuximab	N/A	N/A	N/A	2.82331	0.79765	0.99076

(Not a combination)

resveratrol N/A N/A N/A 6.73193 0.75996 0.99802

(Not a combination)

combination 0.63490 0.67799 0.72447 1.48180 0.75533 0.99537

(2:1)

Drug: cetuximab

Data points entered:

cetuximab µg/mL	Fraction affected
0.2	0.1490
1	0.2403
2	0.3770
4	0.5933
10	0.7127
20	0.8273
40	0.8907
100	0.9543

Median-effect parameters:

	cetuximab µg/mL
Dm:	2.82331
lower 95%:	2.25630
upper 95%:	3.53280

m: 0.79765 +/- 0.044577

y-int: -0.35955 +/- 0.050337

r: 0.99076

CCSI: -0.67605

Drug: resveratrol

Data points entered:

resveratrol µg/mL	Fraction affected
0.1	0.0423
0.5	0.1110
1	0.1813
2	0.2753
5	0.4793
10	0.5970
20	0.6690
50	0.8237

Median-effect parameters:

	resveratrol µg/mL
Dm:	6.73193

lower 95%: 6.05754

upper 95%: 7.48140

m: 0.75996 +/- 0.019556

y-int: -0.62935 +/- 0.018616

r: 0.99802

CCSI: -0.48573

Drug: combination

Combination: cetuximab resveratrol

Ratio: 2:1

Data points entered:

cetuximab (μg/mL)	resveratrol (μg/mL)	Fraction affected
0.2	0.1	0.2210
1	0.5	0.3650
2	1	0.5520
4	2	0.6680
10	5	0.7977
20	10	0.8770
40	20	0.9240
100	50	0.9650

Median-effect parameters:

	cetuximab (μg/mL)	resveratrol (μg/mL)
Dm:	1.48180	0.74090
lower 95%:	1.23566	0.61783
upper 95%:	1.77698	0.88849

m: 0.75533 +/- 0.029762

y-int: -0.129 +/- 0.033607

r: 0.99537

CCSI: -0.67605

Title:

Date:

Investigator:

Filename: Inhibitor-HCT116-CTX+RES.csn

Summary table

Drug	Combination Index Values at					
	ED50	ED75	ED90	Dm	m	r
cetuximab (Not a combination)	N/A	N/A	N/A	103.36417	0.89867	0.96897
resveratrol (Not a combination)	N/A	N/A	N/A	10.90583	0.79597	0.98402
combination (2:1)	0.67483	0.74951	0.83576	12.15445	0.75462	0.99581

Drug: cetuximab

Data points entered:

cetuximab µg/mL	Fraction affected
0.2	0.0077
1	0.0090
2	0.0253
4	0.0397
10	0.0853
20	0.1310
40	0.3767
100	0.6123

Median-effect parameters:

cetuximab

µg/mL

Dm: 103.36417

lower 95%: 51.01586

upper 95%: 209.42801

m: 0.89867 +/- 0.093587

y-int: -1.8103 +/- 0.105680

r: 0.96897

CCSI: -0.67605

Drug: resveratrol

Data points entered:

resveratrol µg/mL	Fraction affected
0.1	0.0270
0.5	0.0830
1	0.1417
2	0.2073
5	0.2610
10	0.3847
20	0.6240
50	0.8453

Median-effect parameters:

resveratrol

µg/mL

Dm: 10.90583

lower 95%: 7.78379

upper 95%: 15.28010

m: 0.79597 +/- 0.058800

y-int: -0.82594 +/- 0.055972

r: 0.98402

CCSI: -0.48573

Drug: combination

Combination: cetuximab resveratrol

Ratio: 2:1

Data points entered:

cetuximab (μg/mL)	resveratrol (μg/mL)	Fraction affected
0.2	0.1	0.0447
1	0.5	0.1313
2	1	0.1880
4	2	0.3530
10	5	0.4370
20	10	0.5587
40	20	0.6893
100	50	0.8550

Median-effect parameters:

	cetuximab (μg/mL)	resveratrol (μg/mL)
Dm:	12.15445	6.07722
lower 95%:	10.45199	5.22600
upper 95%:	14.13420	7.06710

m: 0.75462 +/- 0.028288

y-int: -0.81856 +/- 0.031944

r: 0.99581

CCSI: -0.67605

Title:

Date:

Investigator:

Filename: Inhibitor-HCT116-controlshRNA-CTX+RES.csn

Summary table

Drug	Combination Index Values at					
	ED50	ED75	ED90	Dm	m	r
cetuximab (Not a combination)	N/A	N/A	N/A	89.48251	0.92185	0.97457
resveratrol (Not a combination)	N/A	N/A	N/A	11.82383	0.73784	0.99144
combination (2:1)	0.62158	0.58135	0.55308	11.62647	0.81247	0.99832

Drug: cetuximab

Data points entered:

cetuximab µg/mL	Fraction affected
0.2	0.0073
1	0.0083
2	0.0273
4	0.0407

10 0.0877

20 0.2090

40 0.4223

100 0.5587

Median-effect parameters:

cetuximab

μg/mL

Dm: 89.48251

lower 95%: 48.39723

upper 95%: 165.44580

m: 0.92185 +/- 0.086539

y-int: -1.7992 +/- 0.097720

r: 0.97457

CCSI: -0.67605

Drug: resveratrol

Data points entered:

resveratrol Fraction affected

μg/mL

0.1 0.0327

0.5 0.0817

1 0.1447

2 0.2307

5 0.2710

10 0.4170

20 0.6453

50	0.7717

Median-effect parameters:

	resveratrol
	μg/mL
Dm:	11.82383
lower 95%:	9.20445
upper 95%:	15.18862

m:	0.73784 +/- 0.039675
y-int:	-0.79152 +/- 0.037767
r:	0.99144
CCSI:	-0.48573

Drug: combination

Combination: cetuximab resveratrol

Ratio: 2:1

Data points entered:

cetuximab (μg/mL)	resveratrol (μg/mL)	Fraction affected
0.2	0.1	0.0350
1	0.5	0.1137
2	1	0.1980
4	2	0.3330
10	5	0.4617
20	10	0.5910
40	20	0.7073
100	50	0.8637

Median-effect parameters:

	cetuximab (μg/mL)	resveratrol (μg/mL)
Dm:	11.62647	5.81323
lower 95%:	10.57747	5.28873
upper 95%:	12.77950	6.38975

m: 0.81247 +/- 0.019231

y-int: -0.86564 +/- 0.021716

r: 0.99832

CCSI: -0.67605

Title:

Date:

Investigator:

Filename: Inhibitor-HCT116-shRNA-CTX+RES.csn

Summary table

Drug Combination Index Values at

	ED50	ED75	ED90	Dm	m	r
cetuximab (Not a combination)	N/A	N/A	N/A	91.88506	0.91428	0.97285
resveratrol (Not a combination)	N/A	N/A	N/A	11.89922	0.75090	0.98481
combination (2:1)	0.91546	0.91443	0.92522	17.30464	0.78345	0.99913

Drug: cetuximab

Data points entered:

cetuximab µg/mL	Fraction affected
0.2	0.0073
1	0.0083
2	0.0320
4	0.0437
10	0.0847
20	0.1620
40	0.3840
100	0.6177

Median-effect parameters:

	cetuximab µg/mL
Dm:	91.88506
lower 95%:	48.45274
upper 95%:	174.24948

m: 0.91428 +/- 0.088790

y-int: -1.795 +/- 0.100262

r: 0.97285

CCSI: -0.67605

Drug: resveratrol

Data points entered:

resveratrol Fraction affected

µg/mL

0.1	0.0323
0.5	0.0890
1	0.1397
2	0.1980
5	0.2583
10	0.4030
20	0.5853
50	0.8270

Median-effect parameters:

resveratrol

μg/mL

Dm: 11.89922

lower 95%: 8.50599

upper 95%: 16.64609

m: 0.75090 +/- 0.054041

y-int: -0.80761 +/- 0.051442

r: 0.98481

CCSI: -0.48573

Drug: combination

Combination: cetuximab resveratrol

Ratio: 2:1

Data points entered:

cetuximab (μg/mL)	resveratrol (μg/mL)	Fraction affected
0.2	0.1	0.0300

1	0.5	0.0973
2	1	0.1583
4	2	0.2340
10	5	0.3777
20	10	0.5527
40	20	0.6353
100	50	0.8087

Median-effect parameters:

	cetuximab (μg/mL)	resveratrol (μg/mL)
Dm:	17.30464	8.65232
lower 95%:	16.07303	8.03651
upper 95%:	18.63063	9.31532

m: 0.78345 +/- 0.013379

y-int: -0.97004 +/- 0.015108

r: 0.99913

CCSI: -0.67605

Title:

Date:

Investigator:

Filename: Inhibitor-CT26-CTX+RES.csn

Summary table

Drug Combination Index Values at

	ED50	ED75	ED90	Dm	m	r
cetuximab	N/A	N/A	N/A	70.82971	0.90536	0.97862

(Not a combination)

resveratrol N/A N/A N/A 6.15966 0.77332 0.99820

(Not a combination)

combination 0.72831 0.68269 0.64393 7.64293 0.83076 0.99943

(2:1)

Drug: cetuximab

Data points entered:

cetuximab µg/mL	Fraction affected
0.2	0.0077
1	0.0110
2	0.0467
4	0.0770
10	0.1180
20	0.1737
40	0.4067
100	0.6640

Median-effect parameters:

	cetuximab µg/mL
Dm:	70.82971
lower 95%:	41.68715
upper 95%:	120.34518

m:	0.90536 +/- 0.077691
y-int:	-1.6751 +/- 0.087729
r:	0.97862

CCSI: -0.67605

Drug: resveratrol

Data points entered:

resveratrol µg/mL	Fraction affected
0.1	0.0397
0.5	0.1197
1	0.1897
2	0.2923
5	0.4860
10	0.6350
20	0.6990
50	0.8200

Median-effect parameters:

resveratrol

µg/mL

Dm: 6.15966

lower 95%: 5.57850

upper 95%: 6.80137

m: 0.77332 +/- 0.018993

y-int: -0.61058 +/- 0.018079

r: 0.99820

CCSI: -0.48573

Drug: combination

Combination: cetuximab resveratrol

Ratio: 2:1

Data points entered:

cetuximab (μg/mL)	resveratrol (μg/mL)	Fraction affected
0.2	0.1	0.0490
1	0.5	0.1543
2	1	0.2453
4	2	0.3533
10	5	0.5477
20	10	0.6777
40	20	0.8140
100	50	0.8957

Median-effect parameters:

	cetuximab (μg/mL)	resveratrol (μg/mL)
Dm:	7.64293	3.82147
lower 95%:	7.25445	3.62723
upper 95%:	8.05222	4.02611

m: 0.83076 +/- 0.011423

y-int: -0.73377 +/- 0.012899

r: 0.99943

CCSI: -0.67605

Title:

Date:

Investigator:

Filename: Inhibitor-CT26-controlshRNA-CTX+RES.csn

Summary table

Drug Combination Index Values at

Drug	ED50	ED75	ED90	Dm	m	r
cetuximab	N/A	N/A	N/A	65.78427	0.92959	0.98627
(Not a combination)						
resveratrol	N/A	N/A	N/A	5.73442	0.80503	0.99751
(Not a combination)						
combination (2:1)	0.67878	0.66282	0.65033	6.62911	0.83765	0.99749

Drug: cetuximab

Data points entered:

cetuximab µg/mL	Fraction affected
0.2	0.0063
1	0.0120
2	0.0430
4	0.0857
10	0.1247
20	0.1880
40	0.4030
100	0.6650

Median-effect parameters:

cetuximab

µg/mL

Dm: 65.78427

lower 95%: 43.46488

upper 95%: 99.56476

m: 0.92959 +/- 0.063541

y-int: -1.6901 +/- 0.071751

r: 0.98627

CCSI: -0.67605

Drug: resveratrol

Data points entered:

resveratrol µg/mL	Fraction affected
0.1	0.0407
0.5	0.1117
1	0.1917
2	0.3003
5	0.4897
10	0.6157
20	0.6897
50	0.8697

Median-effect parameters:

resveratrol

µg/mL

Dm: 5.73442

lower 95%: 5.11097

upper 95%: 6.43391

m: 0.80503 +/- 0.023246

y-int: -0.61061 +/- 0.022128

r: 0.99751

CCSI: -0.48573

Drug: combination

Combination: cetuximab resveratrol

Ratio: 2:1

Data points entered:

cetuximab (μg/mL)	resveratrol (μg/mL)	Fraction affected
0.2	0.1	0.0597
1	0.5	0.1647
2	1	0.2543
4	2	0.3680
10	5	0.5543
20	10	0.7027
40	20	0.8407
100	50	0.9137

Median-effect parameters:

	cetuximab (μg/mL)	resveratrol (μg/mL)
Dm:	6.62911	3.31455
lower 95%:	5.94349	2.97174
upper 95%:	7.39382	3.69691

m: 0.83765 +/- 0.024294

y-int: -0.6881 +/- 0.027433

r: 0.99749

CCSI: -0.67605

Title:

Date:

Investigator:

Filename: Inhibitor-CT26-shRNA-CTX+RES.csn

Summary table

Drug	Combination Index Values at					
	ED50	ED75	ED90	Dm	m	r
cetuximab (Not a combination)	N/A	N/A	N/A	68.76697	0.96704	0.97678
resveratrol (Not a combination)	N/A	N/A	N/A	10.97461	0.78020	0.98400
combination (2:1)	0.97954	1.03638	1.11351	16.29815	0.78946	0.99916

Drug: cetuximab

Data points entered:

cetuximab μg/mL	Fraction affected
0.2	0.0053
1	0.0073
2	0.0440
4	0.0750
10	0.1233

20 0.1647

40 0.3780

100 0.6660

Median-effect parameters:

cetuximab

µg/mL

Dm: 68.76697

lower 95%: 39.71363

upper 95%: 119.07489

m: 0.96704 +/- 0.086592

y-int: -1.7768 +/- 0.097781

r: 0.97678

CCSI: -0.67605

Drug: resveratrol

Data points entered:

resveratrol Fraction affected

µg/mL

0.1 0.0323

0.5 0.0760

1 0.1430

2 0.2033

5 0.2633

10 0.4063

20 0.6200

50 0.8410

Median-effect parameters:

	resveratrol
	µg/mL
Dm:	10.97461
lower 95%:	7.82727
upper 95%:	15.38750

m:	0.78020 +/- 0.057669
y-int:	-0.81171 +/- 0.054896
r:	0.98400
CCSI:	-0.48573

Drug: combination

Combination: cetuximab resveratrol

Ratio: 2:1

Data points entered:

cetuximab (µg/mL)	resveratrol (µg/mL)	Fraction affected
0.2	0.1	0.0310
1	0.5	0.0980
2	1	0.1557
4	2	0.2603
10	5	0.4033
20	10	0.5247
40	20	0.6503
100	50	0.8240

Median-effect parameters:

	cetuximab (μg/mL)	resveratrol (μg/mL)
Dm:	16.29815	8.14908
lower 95%:	15.17337	7.58669
upper 95%:	17.50631	8.75316

m: 0.78946 +/- 0.013232

y-int: -0.95694 +/- 0.014942

r: 0.99916

CCSI: -0.67605
